# Sustainable RC Beams with Agricultural Waste Fine Aggregate and Glass Powder: Structural Evaluation under Bending

**DOI:** 10.12688/f1000research.176250.1

**Published:** 2026-01-07

**Authors:** Ziadoon M. Ali, Sheelan Mahmoud Hama, Nahla Hilal, Nebras M. Mhedi, Zouaoui R. Harrat

**Affiliations:** 1 Construction and Projects Department, University of Anbar, Ramadi, Anbar, Iraq; 2 Department of Civil Engineering, University of Anbar, Ramadi, Al Anbar Governorate, Iraq; 3 University of Fallujah, Al-Fallujah, Al Anbar Governorate, Iraq; 4 Laboratoire des Structures et Matériaux Avancés dans le Génie Civil et Travaux Publics, Djilllali Liabes University, Sidi Bel Abbes 22000, Algeria

**Keywords:** Sustainable reinforced concrete beams; Agricultural waste fine aggregate; Waste glass powder in concrete; Flexural behavior of RC beams; Shear performance of concrete beams.

## Abstract

**Background:**

This experimental study evaluates the structural behavior of reinforced concrete (RC) beams incorporating walnut shell fine aggregate (WFA) and 15% powdered waste glass (PWG).

**Method:**

Eight simply-supported RC beams were cast and tested in two series: Group 1 (beams with transverse stirrups i.e. flexure-dominated) and Group 2 (beams without stirrups i.e. shear-dominated). Mixes included a control (0% WFA) and three WFA replacement levels (10%, 20%, 30% by volume of sand), with PWG fixed at 15% of cement in all mixes. Tests included 28-day compressive strength, density measurements, and two-point bending load–deflection tests.

**Results:**

Results show that 10% WFA replacement enhanced compressive strength by 3–5%, flexural capacity by ~6%, and ductility by 64%, while reducing density by 6–8%. Higher WFA contents reduced stiffness but improved energy absorption and crack distribution.

**Conclusions:**

The findings confirm that moderate WFA replacement (≤10%) combined with PWG yields eco-efficient RC beams with improved ductility and reduced dead load.

## Introduction

Concrete production is a major contributor to CO _2_ emissions, mainly due to cement and aggregate consumption. ^1^ In response to the increasing environmental concerns, researchers have explored alternative materials, such as agricultural waste and industrial by-products, for use in concrete to reduce its environmental footprint. ^2– 4^ The integration of waste materials in structural members such as RC slabs and beams remains underexplored. Some efforts include using of recycled concrete aggregates in flexural members, ^5^ incorporation of eggshell and plastic waste in some structural application. ^4– 7^

Among various agricultural residues, walnut shell have emerged as a promising partial replacement for fine aggregates due to their lignocellulosic composition and favorable physical properties. ^8, 9^ Microstructural and thermal performance under elevated temperatures were also favorable when walnut shell replaced sand, showing resistance to cracking and improved insulation. ^10^

Flexural and compressive behavior of concrete incorporating walnut shell was studied in multiple works, indicating a moderate strength reduction but significant weight savings and enhanced sustainability. ^11– 13^ Several studies confirm the potential of walnut shell in reducing the density of concrete while maintaining acceptable mechanical strength. ^14– 16^ Optimization techniques have been employed to identify ideal substitution levels of walnut shell, balancing strength and environmental benefits. ^12, 15, 17^ Despite these advancements, limited studies have investigated the structural performance of RC beams incorporating walnut shell aggregate, especially under bending loads.

Use of glass waste in structural concrete beams and its positive contribution to stiffness and load capacity. ^18, 19^ Nonetheless, research on hybrid use of walnut shell and glass powder in reinforced concrete beams remains limited, especially in terms of structural behavior under bending moments, load-deflection characteristics, and failure modes. Waste glass powder (WGP) is another sustainable alternative that can replace cement or fine aggregates due to its high silica content and pozzolanic activity. ^20– 22^ Numerous studies report that WGP can enhance; compressive and flexural strength by filling voids and densifying the matrix. ^19, 21, 24^ Durability properties such as reduced permeability and improved resistance to sulfate attack, ^23, 25^ Thermal and microstructural stability under elevated temperatures. ^20^ PWG also compensates for strength loss caused by agricultural waste use, making it an excellent synergistic additive for hybrid sustainable concrete. ^19^ However, few studies have explored its combined role with organic fine aggregates like walnut shell in reinforced concrete elements.

Although the sustainability potential of both agricultural waste and glass powder in concrete has been well demonstrated individually, their combined effect in RC beams has not been sufficiently studied. In particular:

• There is a lack of experimental data on load-bearing capacity, crack propagation, and mode of failure of RC beams with walnut shell and PWG.
• Structural implications of using organic fine aggregate and pozzolanic glass powder need to be validated under realistic flexural loading conditions.

This study addresses the aforementioned gap by evaluating the flexural behavior of sustainable RC beams incorporating walnut shell as fine aggregate and glass powder as a strength-enhancing additive. The research contributes to the development of eco-efficient structural elements, offering a dual benefit of waste management and carbon footprint reduction in construction.

For all mixes the cement was replaced with 15% glass powder. The selection of 15% glass powder (GP) as a partial replacement for cement in all concrete mixes was based on prior experimental findings that demonstrated its optimal contribution to mechanical and structural performance. Several studies have investigated the influence of PWG content on the strength and behavior of concrete, and consistently reported that 15% replacement yields superior results. Recent reviews have summarized the environmental and mechanical benefits of incorporating glass powder in green concrete systems, emphasizing its pozzolanic and filler effects. ^26^ Yassen et al. ^27^ experimentally investigated the shear behavior of reinforced concrete beams incorporating waste glass powder and concluded that 15% replacement provides the best balance between strength gain and material efficiency. Similarly, Khudair et al. ^28^ conducted an optimization study on self-compacting concrete using varying P contents and identified 15% as the optimum dosage for enhancing workability, compressive strength, and overall performance. Additionally, Ubeid et al. ^29^ confirmed that 15% PWG enhances not only the compressive and flexural strength, but also significantly improves the energy absorption capacity and bond resistance of concrete key indicators of ductility and structural integrity. These findings collectively validate the selection of 15% PWG in the current study as a performance-based and scientifically supported choice to achieve both environmental and mechanical benefits. ^29^

Recent studies on hybrid waste materials in concrete indicate significant environmental and mechanical benefits; however, there remains limited evidence on their combined structural performance in reinforced elements. ^30– 33^ This study uniquely examines RC beams containing both WFA and PWG under bending, filling this gap.

The selection of 15% PWG as cement replacement was based on previous optimization studies ^27– 29^ that identified this level as providing maximum strength and matrix densification. It is anticipated that higher PWG contents could reduce workability and slightly delay hydration, whereas lower contents might yield less pozzolanic benefit.

## Materials and Method

### A. Materials

In this study, locally sourced materials were selected with an emphasis on sustainability and structural performance.

1. Cement: Ordinary Portland Cement (OPC) conforming to Iraqi Standards No. 5/2019 ^34^ was used as the primary binder. To enhance sustainability and reduce cement consumption, part of the cement was replaced with glass powder.
2. Fine Aggregate: Natural river sand, classified as Zone 2 according to Iraqi Specification No. 45/1984, ^35^ was used as the control fine aggregate with specific gravity of 2.63. For the sustainable mixes, a portion of the sand was replaced by WFA, an agricultural waste product.

The walnut shells were first cleaned to remove organic residues, oven-dried at 105 ± 2 °C for 24 hours, and crushed using a jaw crusher into irregular particles. The crushed shells were then sieved through a series of standard sieves (4.75 mm, 2.36 mm, 1.18 mm, and 0.6 mm) to obtain a particle size distribution comparable to that of natural fine aggregate, with a maximum size limited to 10 mm. Particles retained between 0.6–4.75 mm were used in the mixes to ensure proper grading and packing. Its specific gravity was measured at 0.96, with a water absorption capacity of 10%. Walnut shells were used as partial fine aggregate replacements, taking advantage of their lightweight and lignocellulosic composition, which promotes sustainability and reduces concrete density. The procedure of preparing fine WFA is illustrated in Figure 1.

3. Coarse Aggregate: Crushed gravel with a maximum nominal size of 10 mm was used as the coarse aggregate in all concrete mixes.
4. Powdered Waste Glass (PWG): Waste glass was collected, crushed, and ground into a fine powder, then sieved through a No. 200 sieve (75 μm) to ensure uniform particle size. The resulting PWG had a high silica content and exhibited significant pozzolanic activity. As confirmed by ASTM C1240, ^36^ the pozzolanic activity index (PAI) of the glass powder at 28 days was 95.28%, satisfying the requirements for pozzolanic materials. In all mixes, 15% of the cement content was replaced by PWG to enhance mechanical performance and durability while reducing the environmental footprint.
5. Water: Tap water was used for both mixing and curing purposes.

**Figure 1.**
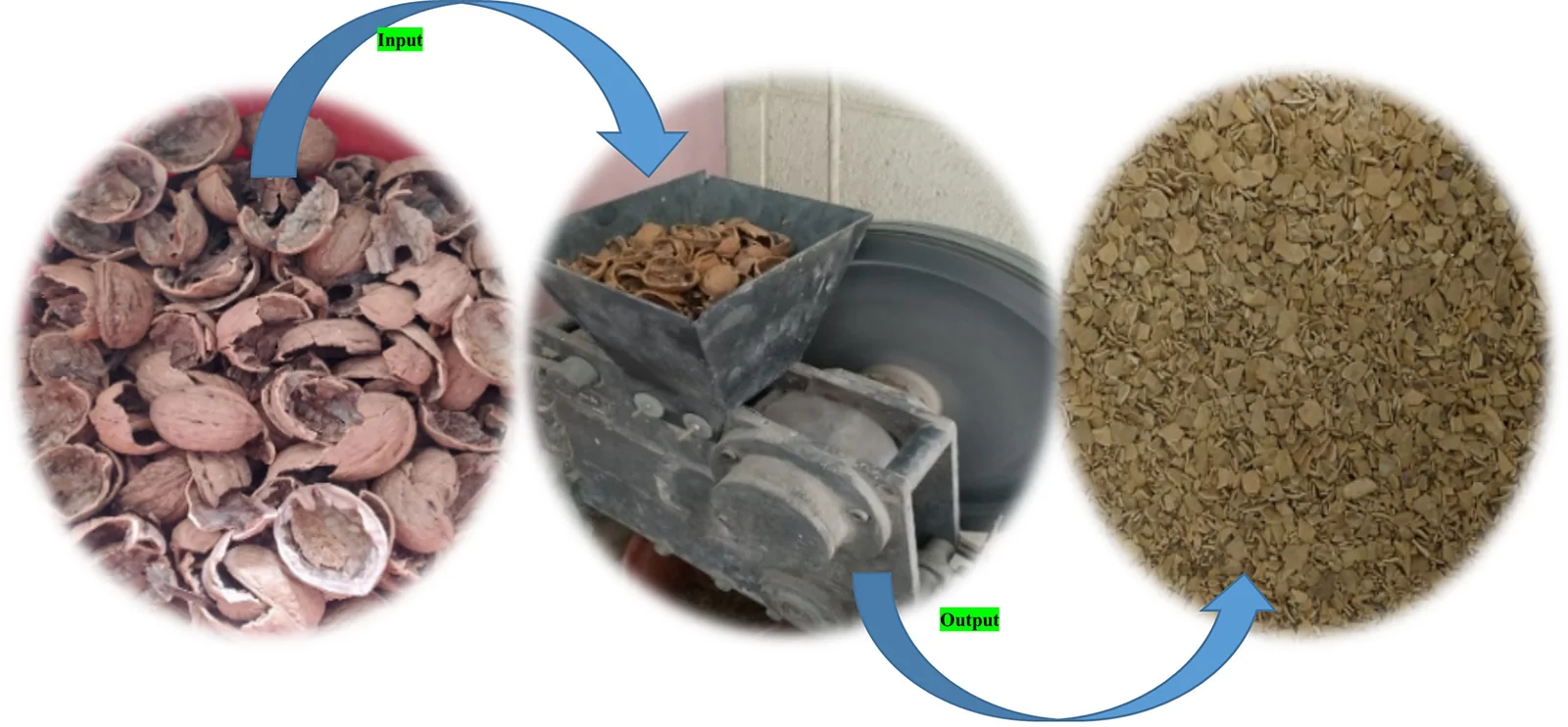
Steps of preparing WFA.

### B. Mix proportion and specimen preparation

The experimental program included four concrete mixes: one control mix using natural fine aggregate (sand) and four sustainable mixes incorporating varying proportions of WFA as partial fine aggregate replacement. In all mixes, 15% of the cement was replaced by PWG by weight. This fixed glass powder content was selected based on previous studies demonstrating its optimal contribution to strength and durability. ^27– 29^ A constant water-to-binder ratio of 0.38 was adopted for all mixtures with 1% superplasticizer (SP). Table 1 presents the detailed mix proportions, including the control mix and the replacement levels of WFA (10%, 20%, and 30%) by volume of fine aggregate.

**Table 1.** Mix proportion in kg/m ^3^.

Mix ID	WFA replacement (%)	Cement	PWG	Water	Superplasticizer	Sand	WFA	Gravel
R	0	405	45	171	4.5	640.0	0.0	1150.0
10%WFA	10	405	45	171	4.5	576.6	23.4	1150.0
20% WFA	20	405	45	171	4.5	511.3	46.7	1150.0
30% WFA	30	405	45	171	4.5	447.0	70.1	1150.0

All constituent materials were measured by weight and mixed in a pan mixer. The mixing process involved the following steps. First, dry mixing of sand, walnut shell (as per the replacement level), cement, and glass powder for 2 minutes. Then gradual addition of water with SP and continued mixing for an additional 3–4 minutes until a uniform consistency was achieved.

A total of eight reinforced concrete beam specimens were cast, grouped into two series (four specimens per group). Each beam had dimensions of 150 mm × 150 mm × 1000 mm and was designed for testing under bending loading. The reinforcement details were kept identical for all beams to ensure consistent comparison.

For group 1, the reinforcement consisted of two longitudinal tension bars of 12 mm diameter (bottom), two compression bars of 6 mm diameter (top), 6 mm diameter stirrups spaced at 55 mm centers along the length of the beam for group 1. While group 2 free from stirrups to check shear strength and the rest reinforcement details is just same as group 1.

After casting, the beams were covered with plastic sheets and kept in the molds for 24 hours. The reinforcing details are shown in Figure 2. Thereafter, all specimens were demolded and cured in water at 23 ± 2°C for 28 days to ensure proper hydration.

**Figure 2.**
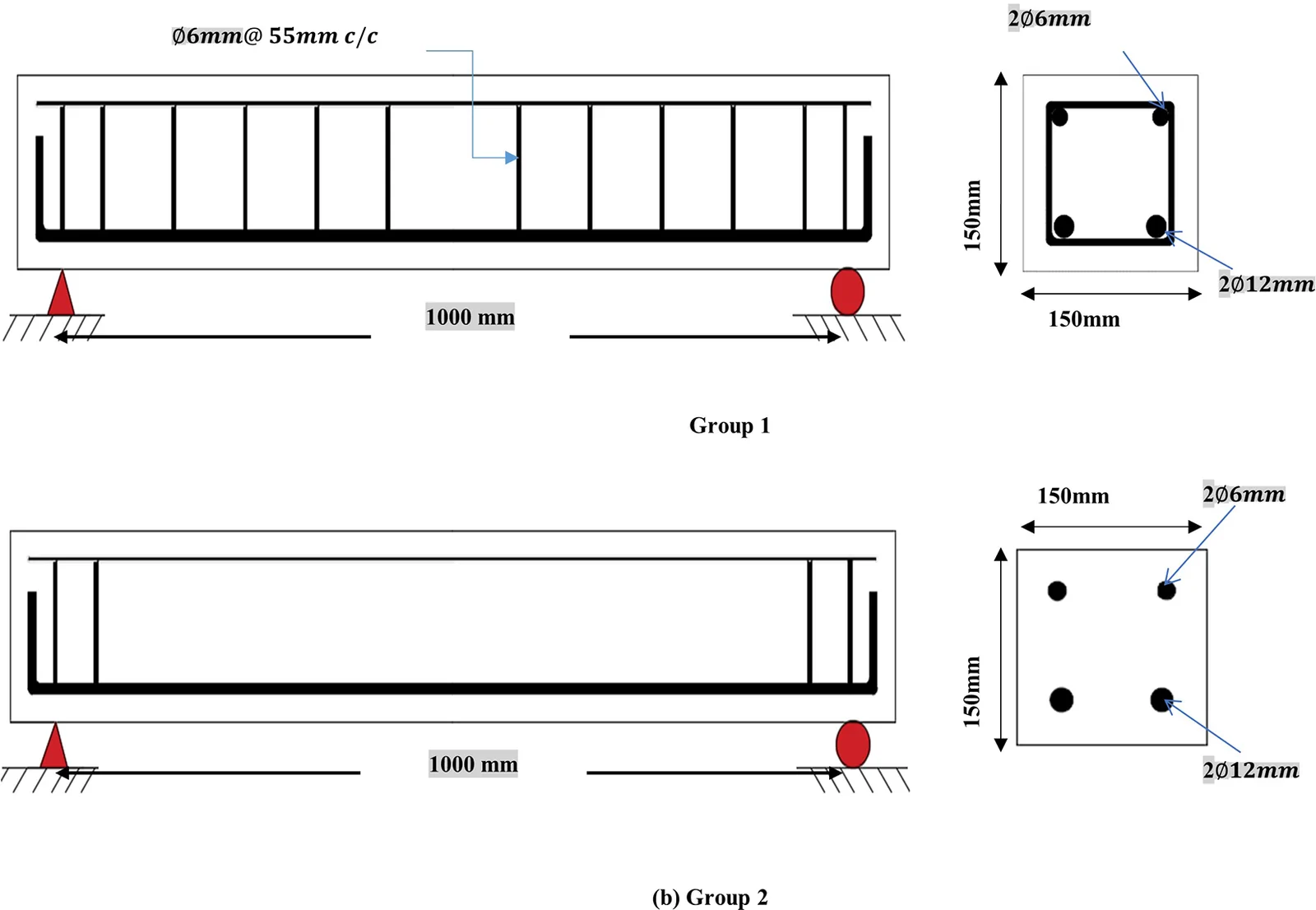
Details of reinforcement.

To evaluate the compressive strength of the concrete mixes, three cube specimens measuring 100 mm × 100 mm × 100 mm were cast for each mix. Also, dry density has been measured.

### C. Test set up

The compressive strength test was performed at 28 days of curing using an ELE Digital Compression Testing Machine with a load capacity of 2000 kN. The loading was applied continuously at a controlled rate until failure. All procedures conformed to the British Standard BS EN 12390-3:2009, ^23^ which outlines the methodology for determining the compressive strength of hardened concrete. The structural performance of the concrete mixes was assessed through flexural testing of eight reinforced concrete beams. Each beam specimen had dimensions of 150 mm × 150 mm × 1000 mm. The bending tests were conducted using a hydraulic jack with a maximum capacity of 500 kN, employing a two-point loading setup to simulate realistic flexural conditions. The load was applied in 5 kN increments, starting from zero and increasing gradually until the beam reached structural failure. A Linear Variable Differential Transformer (LVDT) was installed at the mid-span of each beam to measure the deflection response accurately under increasing load. The LVDT provided real-time displacement data synchronized with the applied load readings. During the testing process, the applied load was continuously recorded using a computer-based data acquisition system. Load-deflection behavior, crack propagation, and failure mode were closely observed and documented. The flexural test setup and instrumentation arrangement are illustrated in Figure 3.

**Figure 3.**
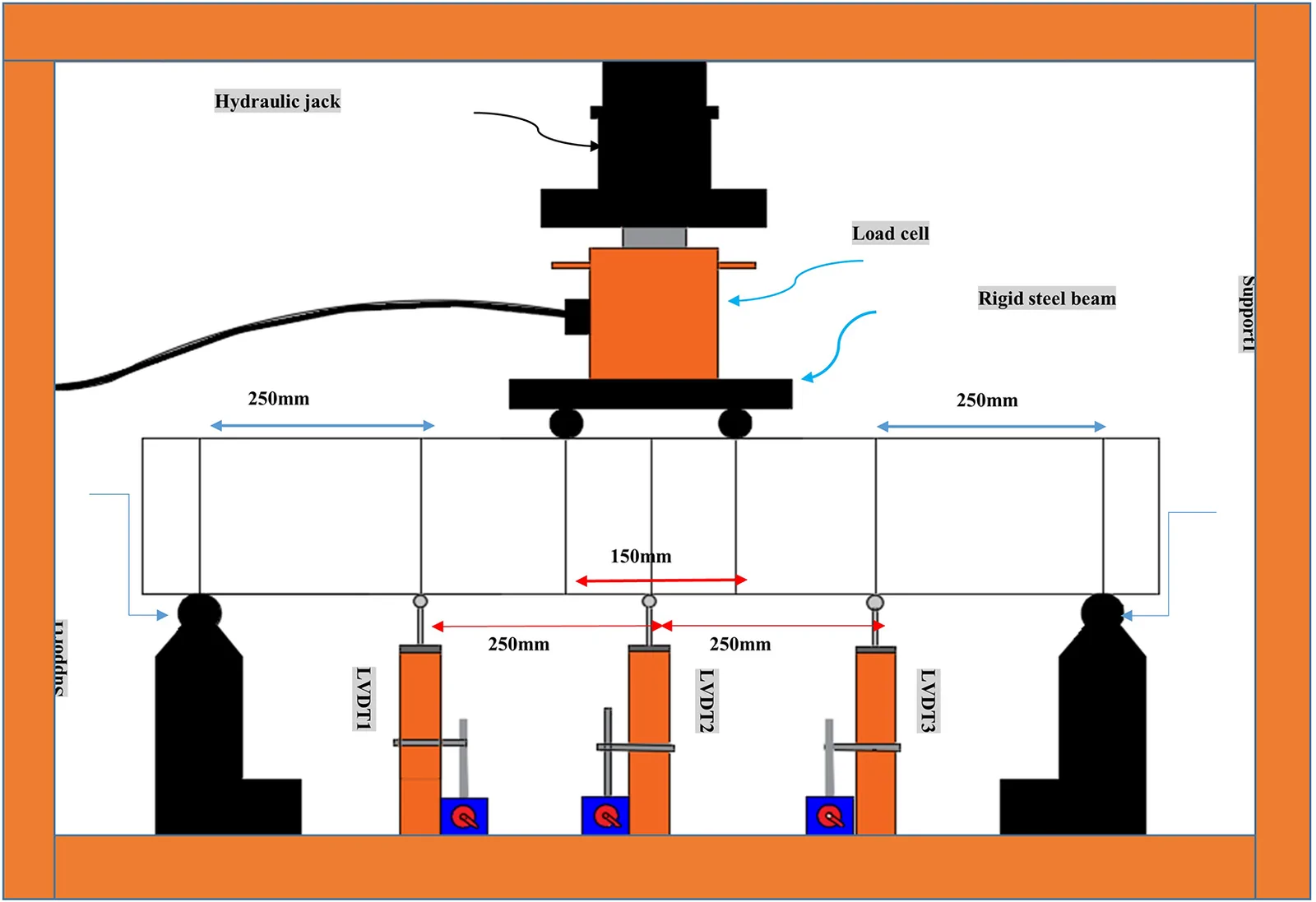
Test set up.

## Results and discussion

### A. Compressive strength of concrete mixes

The 28-day compressive strength results of the concrete mixes are presented in Figure 4. The control mix (RC-0) achieved a compressive strength of approximately 60 MPa, which satisfied the design requirement. At 10% walnut shell replacement (RC-10), a ~3–5% increase in compressive strength was recorded compared with the control. This enhancement can be attributed to the fiber-like morphology and flaky texture of walnut shell particles, which improved crack-bridging and stress transfer within the cementitious matrix. Similar strengthening effects of walnut shell on interfacial bonding and crack resistance were reported by Cheng et al. ^11^ and Hilal et al. ^14^ The presence of 15% pozzolanic waste glass (PWG) in all mixes further contributed to matrix densification and additional hydration reactions, thus counteracting part of the strength loss normally associated with agricultural waste aggregates. ^20, 21^ However, at 20% and 30% replacement levels (RC-20 and RC-30), the compressive strength declined by approximately 8–12% and 15–20%, respectively, compared to the control mix. This reduction is mainly attributed to the lower density, higher porosity, and weaker bonding of walnut shell relative to natural sand. ^8, 16^ Despite these reductions, all mixes maintained compressive strengths within acceptable structural ranges. Comparable findings were reported in other studies that evaluated lignocellulosic waste aggregates. ^12, 15^

**Figure 4.**
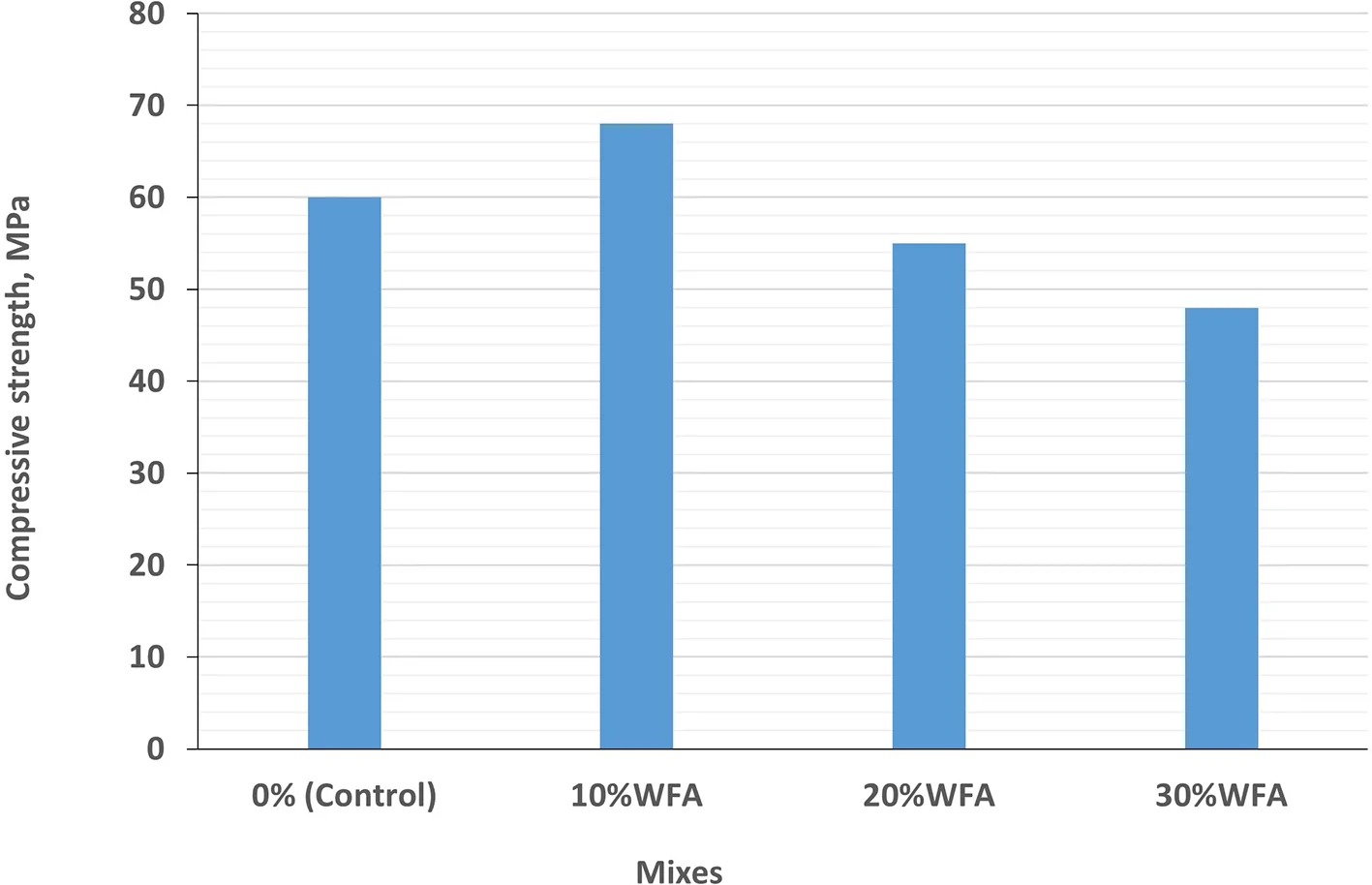
Compressive strength vs. WFA.

### B. Density of concrete mixes

As presented in Figure 5, a progressive reduction in density was observed with increasing levels of walnut shell replacement. The dry density values were consistently lower than the corresponding fresh densities, which is attributed to hydration of cement and the evaporation of free water during drying. The control mix exhibited the highest density of approximately 2400 kg/m³, whereas the incorporation of walnut shell at 10%, 20%, and 30% replacement levels led to decreases of nearly 2%, 4%, and 6–8%, respectively, compared with the control. The lowest density recorded at 30% replacement highlights the intrinsic lightweight and porous structure of walnut shell. ^14, 16^ This density reduction is advantageous in producing lightweight concretes, offering potential applications in both structural and non-structural elements, as also demonstrated in previous studies. ^13^

**Figure 5.**
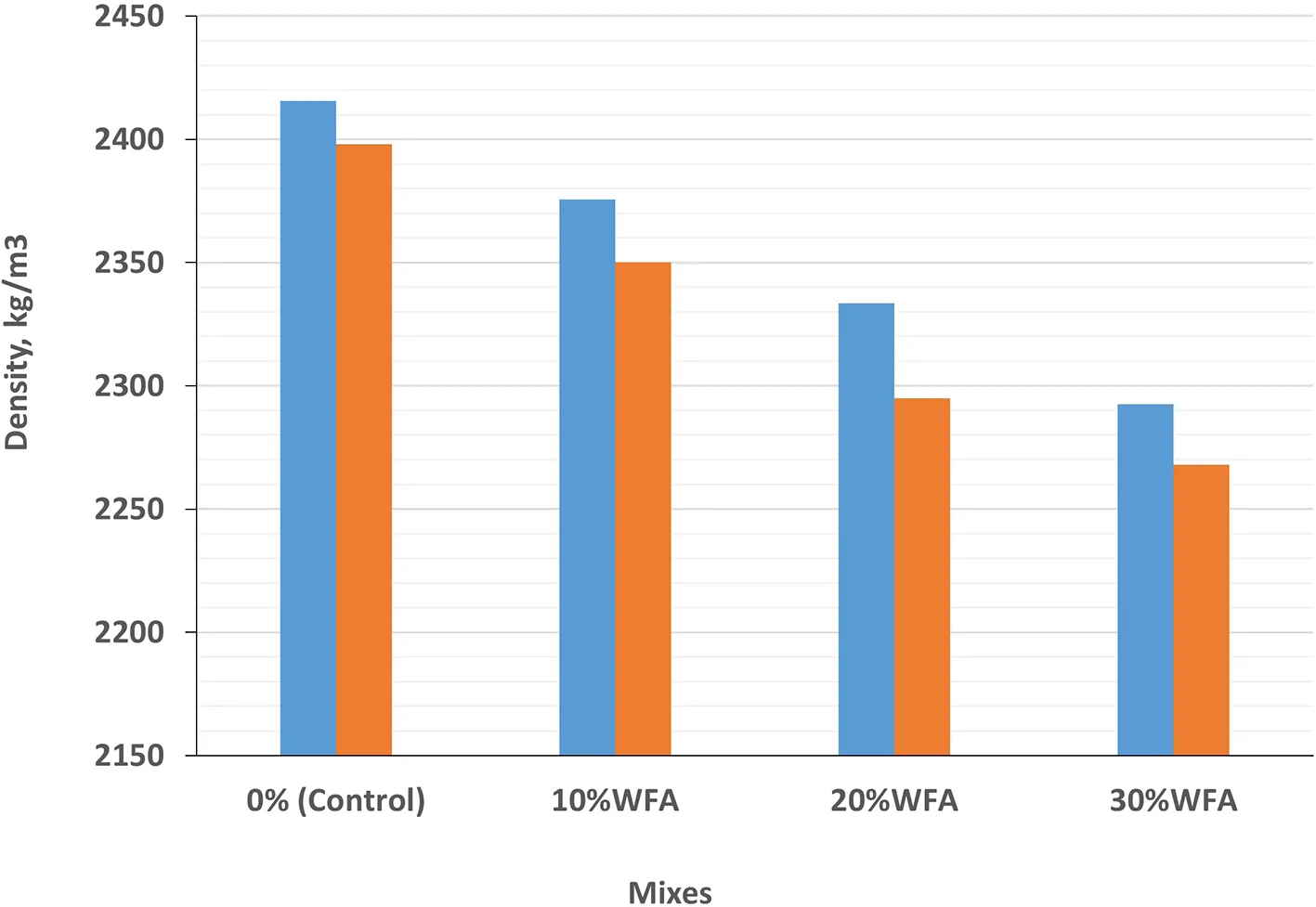
Density vs. WFA.

The combined results demonstrate that partial replacement of fine aggregate with walnut shell up to 10% not only maintained but slightly enhanced compressive strength (~3–5% higher than control), while higher replacement levels led to strength reductions (up to ~20% at 30% replacement) but simultaneously reduced density (~6–8% lighter). These findings suggest that walnut shell can serve as a sustainable lightweight aggregate in structural concrete when used at moderate levels, particularly when combined with pozzolanic waste glass to compensate for potential strength losses. This aligns with current research directions aimed at developing environmentally friendly and resource-efficient concrete. ^3, 19, 23, 25^

### C. Load–deflection behavior

• **Flexural Load–Deflection Behavior (Group 1)**

The curves in Figure 6 show that beams with 10% WFA replacement achieved higher ultimate load capacity and greater ductility compared with the control beam. This indicates that a limited amount of WFA enhances the post-cracking behavior of beams, acting somewhat like discrete fibers that improve energy absorption and crack bridging. However, at 20% and 30% replacement, the load capacity and stiffness decreased. This reduction is consistent with the decline in compressive strength at higher WFA content, mainly due to the porous and organic nature of WFA, which weakens the matrix continuity and reduces aggregate interlock. Even so, these higher replacement levels still maintained reasonable ductility, which is advantageous for applications requiring lightweight materials with better energy dissipation capacity.

**Figure 6.**
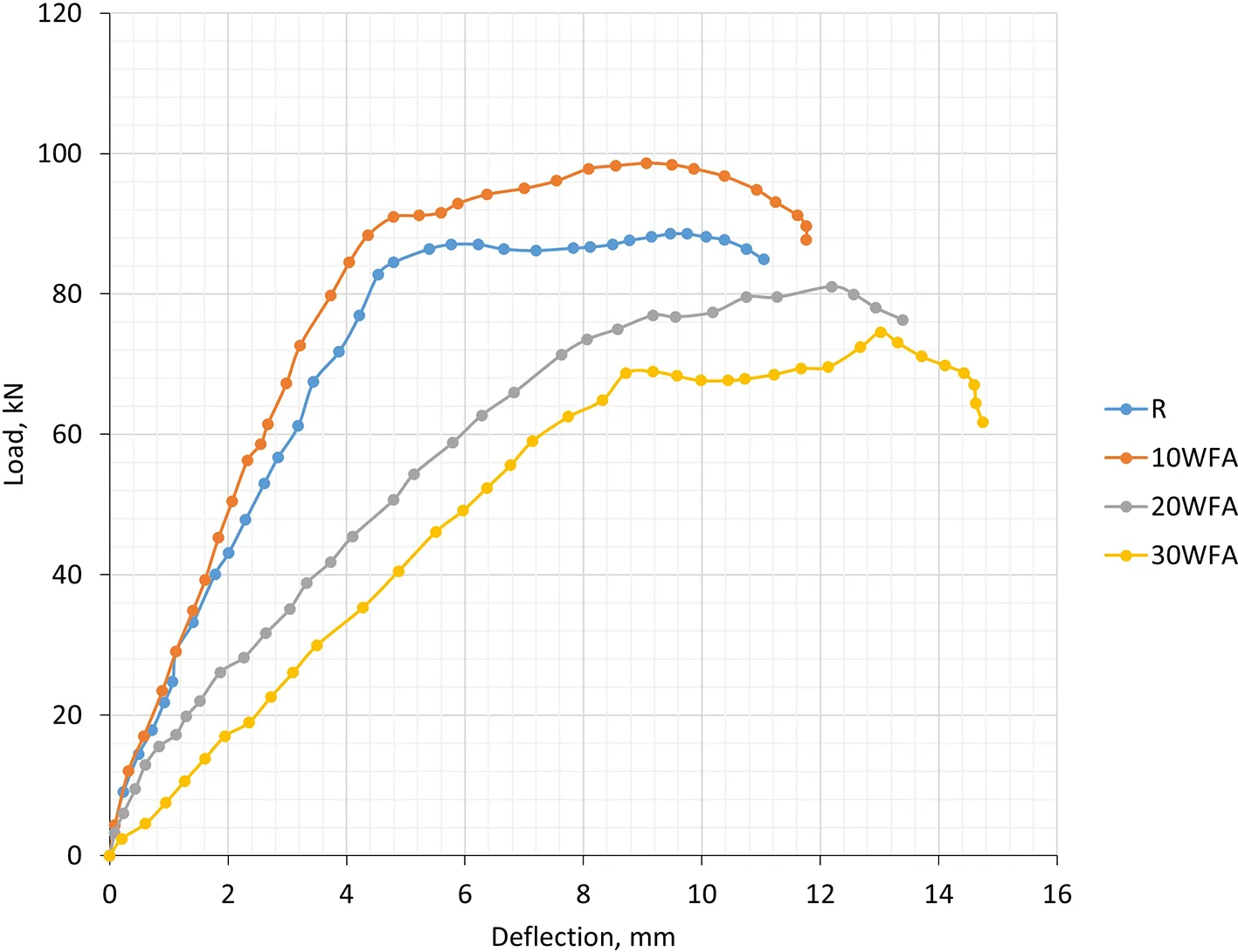
Flexural Load–Deflection Behavior (Group 1).

The addition of glass powder across all mixes refined the pore structure and improved the bond between cement paste and aggregate, which reduced brittleness and contributed positively to flexural performance.

• **Shear Load–Deflection Behavior (Group 2)**

Beams in this group failed predominantly in shear rather than flexure. The curves in Figure 7 show a sharper load drop after peak load compared to flexural failure, reflecting the more brittle nature of shear failure. With increasing WFA content (10%, 20%, 30%), there is a general reduction in peak load capacity, similar to the flexural group. However, the deflection at failure increased slightly with higher WFA content, suggesting some improvement in ductility even under shear-dominated behavior. The presence of glass powder again contributed to improved crack control and bond strength, mitigating the suddenness of shear failure to some extent.

**Figure 7.**
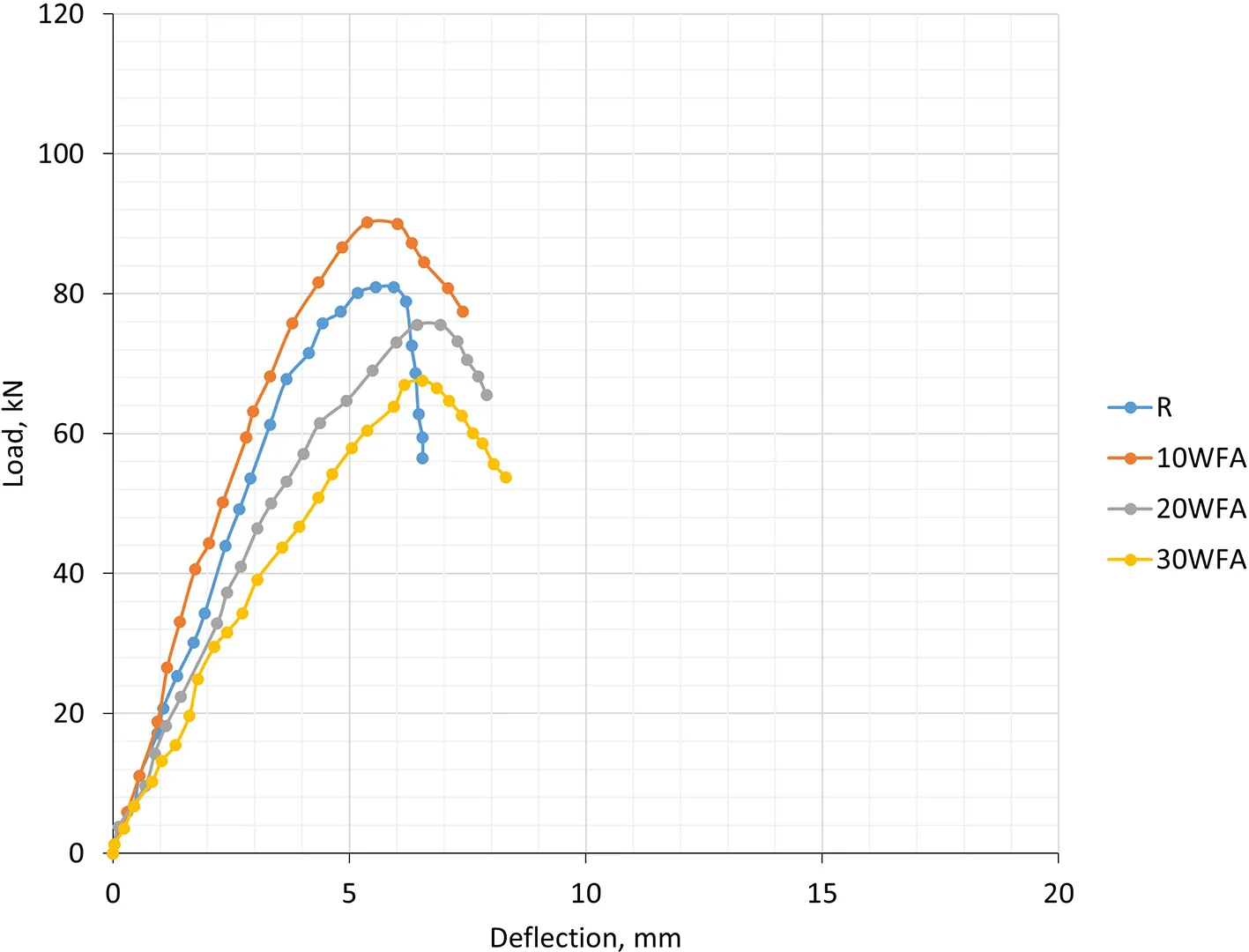
Shear Load–Deflection Behavior (Group 2).

### D. Crack patterns and failure modes

• **Flexural Behavior-Group 1**

Crack propagation and failure modes are illustrated in Figure 8. All beams failed in flexural tension, with initial cracks forming at mid-span and progressing toward the compression zone as loading increased. The control beam showed fewer, more localized vertical cracks, indicative of a brittle failure mode. The 10% WFA beam exhibited wider but more distributed cracks, reflecting enhanced ductility and better energy dissipation capacity. Beams with 20% and 30% WFA displayed multiple fine cracks along the span, and the failure occurred more gradually a hallmark of ductile behavior, likely promoted by the combined effect of glass powder and the flexible nature of the walnut shell. These results reinforce that 10% WFA is an optimum replacement level, providing performance improvements in both compressive and flexural behavior, while higher WFA contents offer benefits in terms of lightweight characteristics and ductility, albeit with a trade-off in strength.

• **Shear Behavior-Group 2**

**Figure 8.**
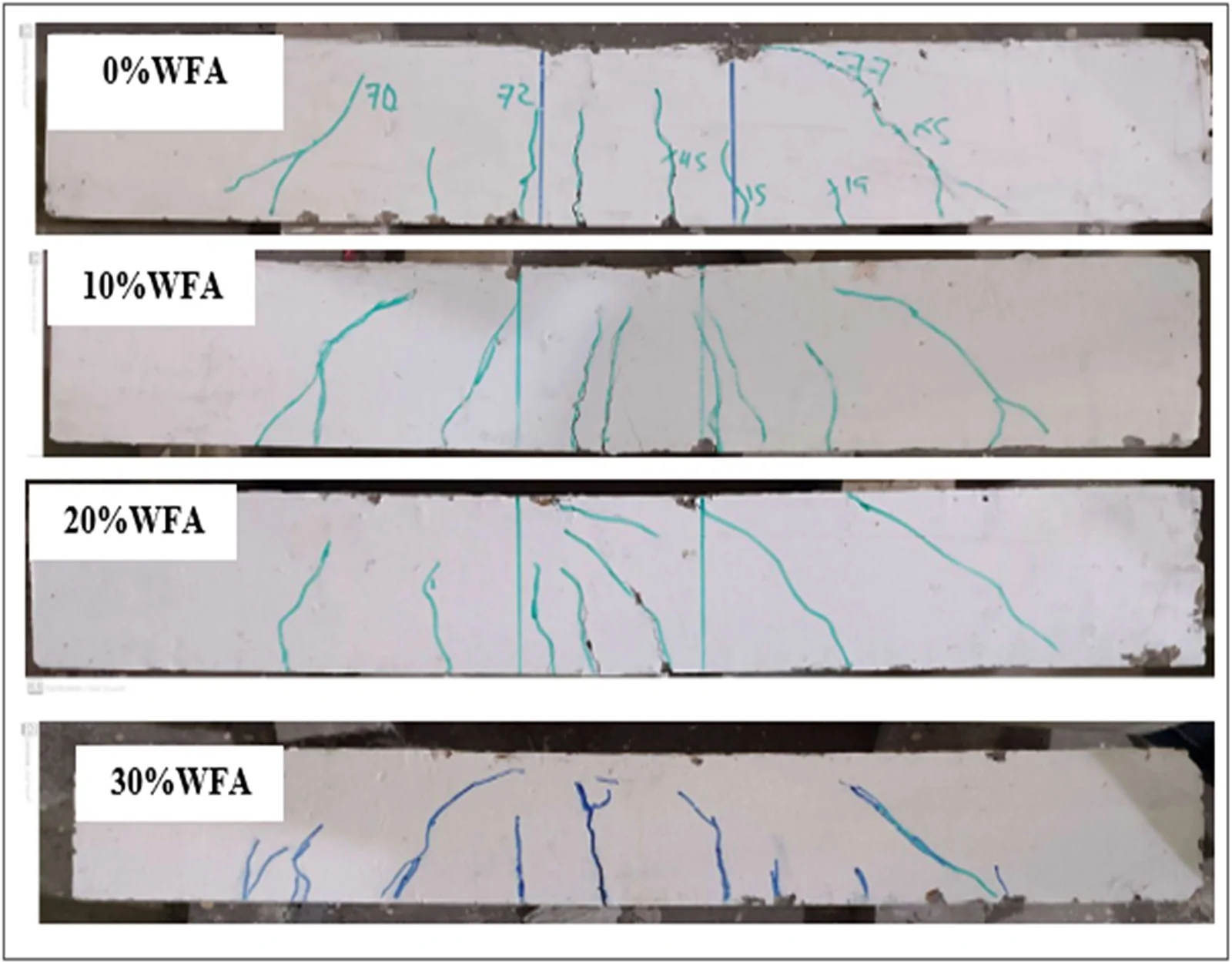
Crack pattern (Group 1).

In Group 2, all beams were tested without transverse stirrups to intentionally provoke shear-governed failure, see Figure 9. The observed cracking patterns and failure mechanisms are consistent with diagonal shear cracking and sudden shear-slip mechanisms typical of lightly reinforced beams lacking shear reinforcement.

**Figure 9.**
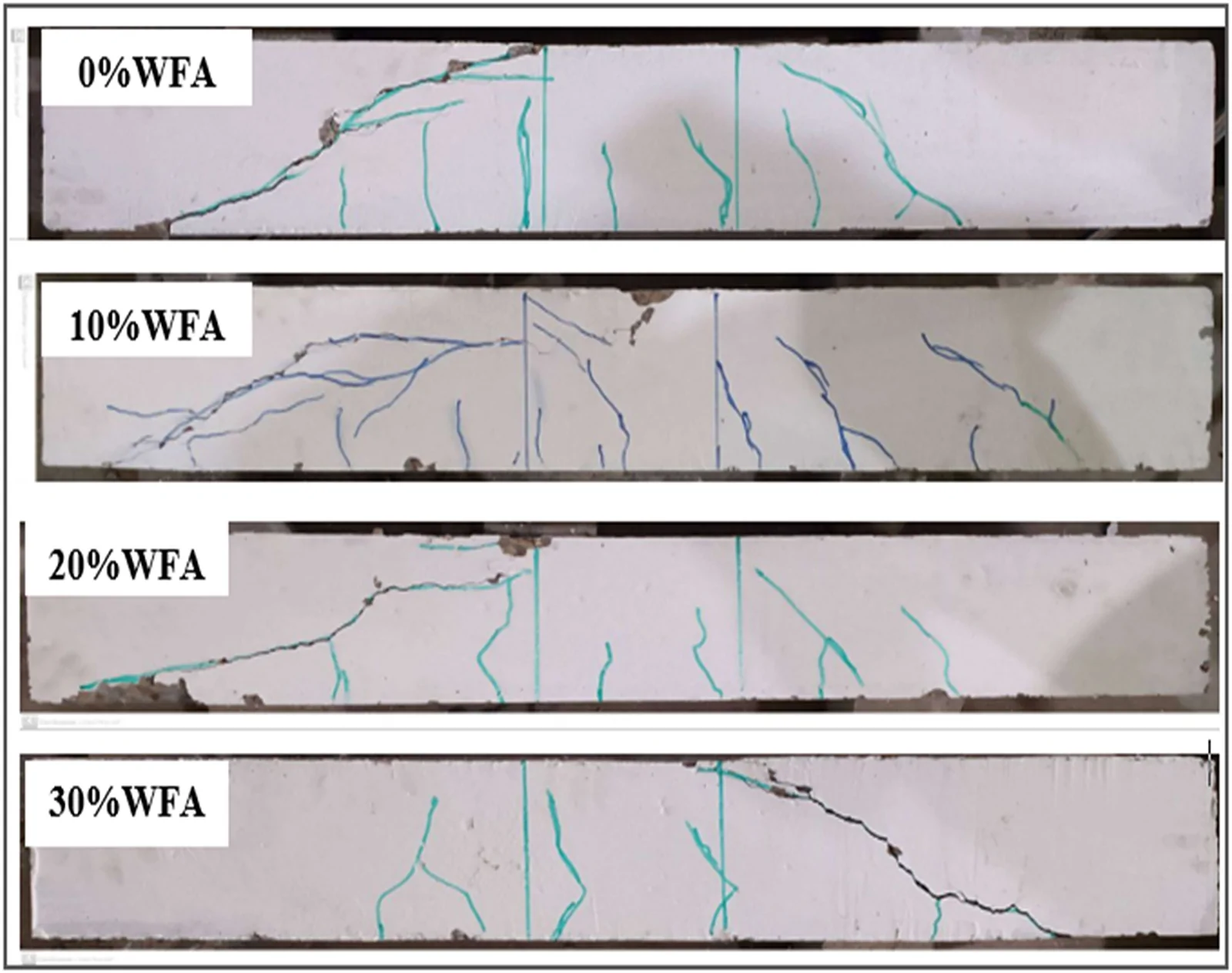
Crack pattern (Group 2).

The first visible cracks formed near the expected critical shear span (approximately at the support region and along the shear span between the loading point and support). Initial cracks were predominantly inclined (diagonal) relative to the beam axis and developed at relatively low load levels, indicating the early development of principal tensile stresses due to combined shear and bending. With increasing load the inclined cracks widened and propagated rapidly toward the compression zone and adjacent support regions. In the control beam, diagonal cracks were fewer but coarser and rapidly progressed to a single dominant diagonal shear crack, culminating in a brittle drop in load. In WFA-containing beams, cracks tended to be more distributed: a network of multiple diagonal micro-cracks coalesced prior to formation of the dominant crack. This distribution was more pronounced with higher WFA contents (20–30%), likely related to reduced stiffness and increased deformability of the matrix and weaker aggregate interlock.

All Group 2 beams failed by diagonal shear characterized by sudden propagation of the principal diagonal crack connecting the load application zone to the support. The failure in the control beam occurred more abruptly, with a clean diagonal fracture plane and limited post-peak load capacity. In contrast, WFA mixes exhibited a slightly more progressive failure: although the peak load was lower, the post-peak decay of load was less instantaneous in mixes with PWG, and the crack pattern near failure included additional secondary splits and localized crushing at the compression zone adjacent to the diagonal crack. At higher WFA replacements (20–30%), the diagonal cracks were accompanied by more pronounced sliding and wider crack openings, indicating reduced aggregate interlock and frictional resistance along the crack plane.

Across WFA percentages, the presence of 15% PWG improved matrix cohesion and limited crack width growth in the tension face, delaying the abruptness of full shear collapse. PWG appears to refine the microstructure and improve bond, producing finer and more numerous cracks rather than a single catastrophic fracture surface. This effect mitigated the brittleness of shear failure to some degree, though it did not fully compensate for the reduction in shear capacity caused by increased WFA content. The observed behavior indicates that while moderate WFA replacement (≤10%) maintains shear performance close to the control and offers benefits in ductility and energy absorption, higher replacements (≥20%) significantly reduce shear capacity and increase the risk of brittle shear failure. For structural applications where shear governs, the use of WFA should be limited or accompanied by adequate shear reinforcement (stirrups) and/or shear-strengthening measures.

### E. Toughness, ductility, and stiffness indexes

The evaluation of toughness, ductility, and stiffness indices provides an in-depth understanding of the overall structural efficiency of reinforced concrete (RC) beams incorporating WFA and PWG. These indices move beyond peak strength alone, offering insights into the energy absorption capacity, deformation behavior, and load–deflection response of sustainable beams under both flexural and shear-dominated conditions. The results are illustrated in Table 2 and Figure 9.

**Table 2.** Beam performance indexes.

Beam ID	Group	Toughness T (kN·mm)	Ductility μ (Δu/Δy)	Initial stiffness k (Pcr/Δcr) (kN/mm)
R	1	551.0	3.00	22.5
10WFA	1	662.6	4.93	32.0
20WFA	1	606.6	3.27	15.64
30WFA	1	590.3	3.50	8.89
R	2	269.9	1.76	19.23
10WFA	2	347.7	1.79	24.0
20WFA	2	273.1	2.29	12.8
30WFA	2	293.0	2.18	11.38

**1. Toughness**

Toughness, measured as the area under the load–deflection curve, reflects the total energy absorption before failure. The results are shown in Figure 10a.

**Figure 10.**
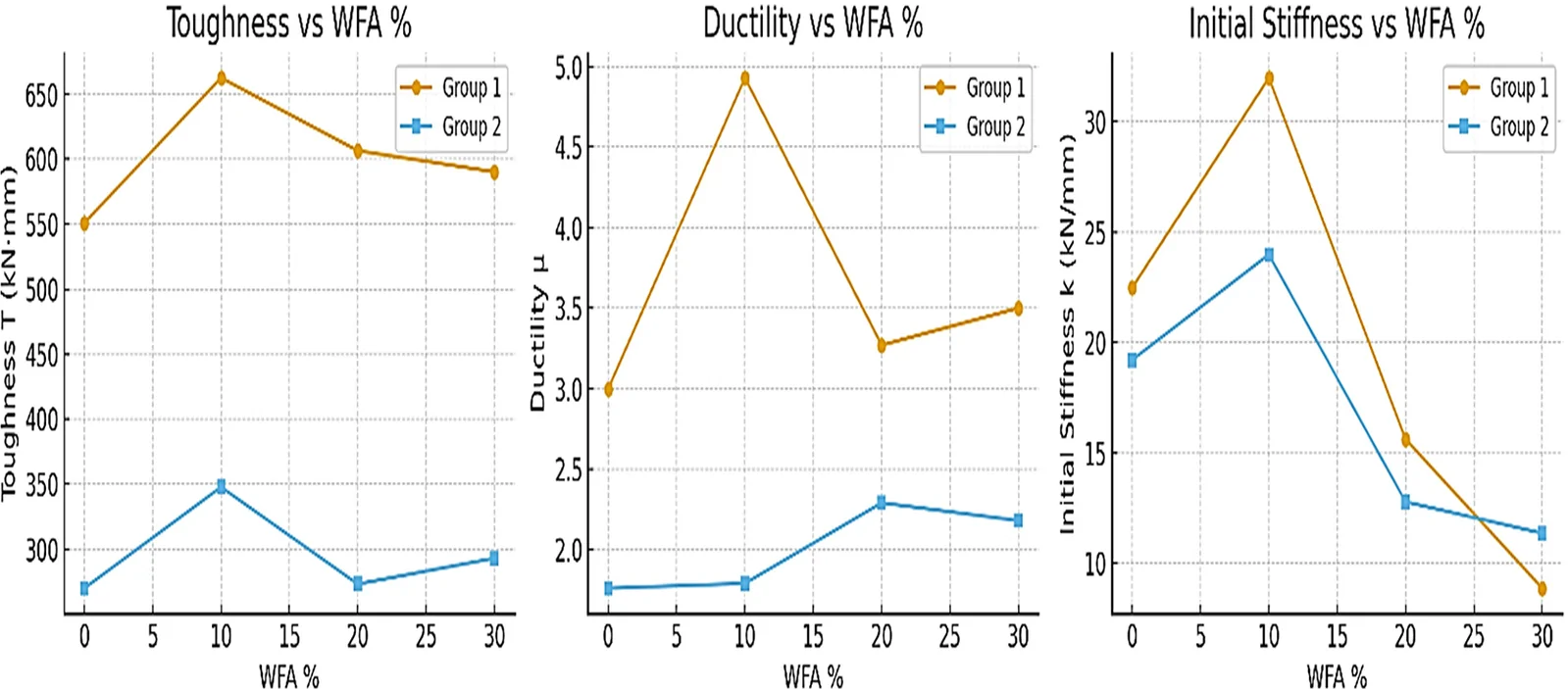
Toughness, ductility, and stiffness indexes vs. WFA.

For flexure-dominated beams (Group 1), the control beam absorbed 551 kN·mm, while the 10% WFA beam reached the highest toughness (662.6 kN·mm), reflecting a 20% improvement. This enhancement demonstrates the crack-bridging role of WFA particles and the matrix refinement from PWG, both of which delay catastrophic cracking and enable greater energy dissipation. At 20% and 30% WFA replacement, toughness values were 606.6 kN·mm (+10.1%) and 590.3 kN·mm (+7.1%), respectively, showing that even higher WFA levels preserved or slightly improved toughness compared to control. This behavior indicating that even at higher replacement levels, beams retain significant energy absorption capacity despite reduced stiffness and peak load. In shear-dominated beams (Group 2), overall toughness values were much lower due to the inherently brittle nature of shear failure. Nevertheless, 10% WFA increased toughness by nearly 29% compared to the control (347.7 vs. 269.9 kN·mm), again highlighting its effectiveness in delaying brittle collapse. At 20% and 30% WFA, toughness values were 273.1 kN·mm (+1.2%) and 293.0 kN·mm (+8.6%), indicating marginal but consistent improvements. At higher WFA levels, toughness remained slightly above or comparable to the control, suggesting that WFA contributes to spreading micro-cracks and distributing shear stresses more evenly, even though the ultimate shear strength decreases. Thus, toughness improvements were most pronounced at 10% WFA, with benefits extending even to shear-dominated beams that typically exhibit brittle failure.

**2. Ductility**

The ductility index (μ = Δu/Δy) reflects the deformation capacity beyond yielding, which is a crucial safety parameter, especially for seismic applications. The results of ductility are shown in Figure 10b.

In flexural beams, ductility improved significantly at 10% WFA (μ = 4.93), representing a ~64% increase relative to the control (μ = 3.0). This demonstrates the ability of the sustainable mix to undergo larger deflections without sudden failure, a desirable trait for structural resilience. At 20% and 30% replacement, ductility remained above the control (μ = 3.27 and 3.50), confirming that higher WFA levels enhance deformability even though they reduce strength and stiffness.

For shear-dominated beams, ductility was much lower overall due to the abrupt nature of Diagonal shear cracking. The control beam recorded μ = 1.76, while 10% WFA showed only a marginal improvement (μ = 1.79). Interestingly, higher WFA levels (20% and 30%) resulted in improved ductility (μ = 2.29 and 2.18), suggesting that although shear strength declined, the distributed cracking promoted by WFA allowed a more gradual failure process. ^37^

**3. Initial Stiffness**

Initial stiffness (k = Pcr/Δcr) is a measure of the beam’s elastic rigidity prior to cracking. It is acknowledged that determining the exact first-cracking load may involve minor uncertainty, as micro-cracks can develop before visible cracking is observed. Therefore, Pcr was defined as the load at which the first visible surface crack appeared and a noticeable deviation from linearity occurred on the load–deflection curve. This practical definition aligns with standard flexural testing procedures and provides a consistent basis for comparing the stiffness of different beam mixes.

For Group 1 beams, stiffness increased markedly at 10% WFA (32.0 kN/mm vs. 22.5 kN/mm for control), showing that moderate WFA replacement combined with PWG produces a stiffer and stronger matrix. However, at 20% and 30% WFA, stiffness dropped significantly (15.64 and 8.89 kN/mm), consistent with the reduced compressive strength and increased porosity at higher replacement levels. In Group 2 beams, a similar pattern was observed. The control beam exhibited a stiffness of 19.23 kN/mm, while the 10% WFA beam showed improvement (24.0 kN/mm). At 20% and 30% replacement, stiffness declined (12.8 and 11.38 kN/mm), reflecting the weaker aggregate interlock of WFA and reduced crack resistance in shear. The combined analysis indicates that 10% WFA replacement offers the most favorable balance across all indices: increased toughness, markedly improved ductility, and enhanced stiffness, both in flexural and shear behavior. This confirms the synergistic role of WFA and PWG in producing beams with superior energy dissipation and resilience. Higher WFA contents (20–30%) reduce stiffness and peak strength but improve ductility and crack distribution, making them suitable for lightweight or seismic-resistant applications where deformability is prioritized over load capacity.

The reduction in stiffness with higher WFA content can be attributed to the porous and lightweight nature of walnut shells, which decreases aggregate interlock and elastic modulus. ^8, 16^ Conversely, PWG refines the cement matrix and improves bond integrity, contributing to the observed increase in cracking resistance and ductility.

## Conclusions

1. Incorporating 10% WFA as a sand replacement enhanced both compressive and flexural strength, attributed to its fiber-like role in bridging cracks and improving post-cracking resistance. At higher replacement levels (20–30%), a gradual reduction in strength was observed; however, ductility and cracking performance were significantly improved.
2. The use of PWG was for enhanced the homogeneity and compactness of the matrix because its pozzalanic effect, which likely contributed to better stress transfer and crack control.
3. Beams with 10% WFA replacement demonstrated the most favorable balance between strength and ductility, confirming their suitability for structural applications where flexural performance is a governing factor. PWG enhanced the homogeneity and compactness of the matrix.
4. Increasing WFA content beyond 10% reduced stiffness and ultimate load capacity but enhanced ductility, highlighting its potential in applications that prioritize lightweight construction, seismic energy dissipation, and improved deformability.
5. In shear-dominated behavior, walnut shell replacement reduced shear capacity due to weaker aggregate interlock; nevertheless, it improved energy absorption and delayed brittle shear failure, enhancing overall structural resilience.
6. At low replacement levels (≤10%), the shear behavior of WFA beams remained comparable to the control mix, whereas higher replacement levels led to reduced shear strength but preserved improved ductility.
7. For beams where shear strength is critical, WFA replacement should be limited to moderate levels (≤10%). Conversely, higher replacement levels can be adopted in scenarios where dead load reduction, ductility, and sustainability are prioritized over peak strength capacity.
8. The density reduction achieved through WFA replacement directly contributes to lowering the dead load of concrete members. At 30% replacement, reductions of up to 6–8% in density were observed, offering advantages in structural efficiency, foundation design, and overall material savings.
9. Overall, the 10% WFA replacement mix (10 WFA) provided optimal performance, combining strength, ductility, and sustainability. This mix is recommended for structural applications aiming to balance mechanical efficiency, dead load reduction, and environmental benefits.

The findings presented are valid within the experimental scope of this study, which involved small-scale beams and fixed PWG content (15%). Future investigations on full-scale members and varied PWG levels are necessary to generalize these conclusions for design applications.

## Data Availability

The datasets supporting the finding of this study are openly available in Zenodo repository at https://doi.org/10.5281/zenodo.18057324. ^38^ This project contains the following underlying data:
•
the data avialable.xlsx Data are available under the terms of the Creative Commons Attribution 4.0 International license (CC-BY 4.0).
