# Supplementary material for: Sustainable RC Beams with Agricultural Waste Fine Aggregate and Glass Powder: Structural Evaluation under Bending
Source: F1000Res. 2026 Oct 16;15:20. Originally published 2026 Jan 7. [Version 4] doi: 10.12688/f1000research.176250.4 (PMC13695713; doi:10.12688/f1000research.176250.4)
Supplement: Peer review history

# Reviewer report: Reviewer response for version 4

**DOI:** 10.5256/f1000research.212532.r530657

**Published:** 2026-10-27

**Authors:** Piotr Smarzewski

**Affiliations:** 1 Military University of Technology, Warsaw, Poland

The manuscript has been improved following the previous review rounds, and the authors have addressed several concerns. However, some limitations remain, particularly the lack of quantitative comparison with design-code predictions, residual numerical inconsistencies, and insufficiently supported interpretations of the PWG contribution.

 Considering the improvements made and the limitations of the experimental program, I recommend approval with reservations.

Is the work clearly and accurately presented and does it cite the current literature?

Yes

If applicable, is the statistical analysis and its interpretation appropriate?

Not applicable

Are all the source data underlying the results available to ensure full reproducibility?

Partly

Is the study design appropriate and is the work technically sound?

Partly

Are the conclusions drawn adequately supported by the results?

Partly

Are sufficient details of methods and analysis provided to allow replication by others?

Partly

Reviewer Expertise:

Structural engineering; concrete technology; fiber-reinforced concrete; high-performance and self-compacting concrete; sustainable cementitious composites; structural behavior of reinforced concrete elements.

I confirm that I have read this submission and believe that I have an appropriate level of expertise to confirm that it is of an acceptable scientific standard, however I have significant reservations, as outlined above.

# Reviewer report: Reviewer response for version 4

**DOI:** 10.5256/f1000research.212532.r530656

**Published:** 2026-10-24

**Authors:** Yasin Onuralp Özkılıç

**Affiliations:** 1 Necmettin Erbakan University, Konya, Turkey

Paper can be accepted

Is the work clearly and accurately presented and does it cite the current literature?

No

If applicable, is the statistical analysis and its interpretation appropriate?

No

Are all the source data underlying the results available to ensure full reproducibility?

No

Is the study design appropriate and is the work technically sound?

No

Are the conclusions drawn adequately supported by the results?

Yes

Are sufficient details of methods and analysis provided to allow replication by others?

No

Reviewer Expertise:

Civil Engineering

I confirm that I have read this submission and believe that I have an appropriate level of expertise to confirm that it is of an acceptable scientific standard.

# Reviewer report: Reviewer response for version 3

**DOI:** 10.5256/f1000research.208410.r519851

**Published:** 2026-09-22

**Authors:** Piotr Smarzewski

**Affiliations:** 1 Military University of Technology, Warsaw, Poland

The manuscript has been improved. However, several essential issues still need to be addressed before it can be considered acceptable.

 1. The structural analysis of the beams remains too descriptive. The discussion is still based mainly on load–deflection curves, percentage changes, toughness, ductility and stiffness indices. At least a basic comparison between the experimentally obtained flexural/shear capacities and predictions from an established design approach should be added. Otherwise, the paper remains primarily a comparison of experimental results rather than a structural evaluation of RC beams. This issue was also identified previously during peer review.

 2. The authors should clearly acknowledge that only one beam was tested for each WFA level within each group. Eight beams were tested in total, with four specimens in each group. Therefore, statements implying statistical significance, repeatability or an experimentally proven “optimum” WFA content should be avoided. The conclusions should refer to trends observed in the tested specimens.

 3. The role of PWG is overstated. Since 15% PWG was used in all mixtures, including the reference mixture, the present experimental design cannot isolate the effect of PWG or demonstrate a synergistic interaction between PWG and WFA. Statements claiming that PWG experimentally improved matrix densification, crack control or structural response should therefore be reformulated as literature-supported explanations or hypotheses rather than findings demonstrated by this experiment.

 4. The definition of ductility must be clarified. The procedure used to determine the yield displacement is not sufficiently described. The authors should specify exactly how the yield displacement was determined and avoid interpreting this index as true structural ductility if yielding was not verified.

 5. The numerical inconsistencies must be corrected throughout the manuscript. For example, the reported increase in compressive strength at 10% WFA is inconsistent between the Abstract and Results, and the reported density reductions are also inconsistent between the Results and Conclusions. All percentage changes should be recalculated directly from the reported experimental values and made consistent throughout the manuscript.

 6. The conclusions should be moderated. Claims concerning structural suitability, seismic-resistant applications, synergy and “optimal performance” go beyond what can be demonstrated using eight small-scale beams and one specimen per configuration. The conclusions should therefore be restricted to the observed experimental trends.

Is the work clearly and accurately presented and does it cite the current literature?

Yes

If applicable, is the statistical analysis and its interpretation appropriate?

Not applicable

Are all the source data underlying the results available to ensure full reproducibility?

Partly

Is the study design appropriate and is the work technically sound?

Partly

Are the conclusions drawn adequately supported by the results?

Partly

Are sufficient details of methods and analysis provided to allow replication by others?

Partly

Reviewer Expertise:

Structural engineering; concrete technology; fiber-reinforced concrete; high-performance and self-compacting concrete; sustainable cementitious composites; structural behavior of reinforced concrete elements.

I confirm that I have read this submission and believe that I have an appropriate level of expertise to confirm that it is of an acceptable scientific standard, however I have significant reservations, as outlined above.

# Response

**Published:** 2026-10-09

**Authors:** Sheelan Hama

**Affiliations:** Civil Engineering, University of Anbar, Ramadi, Al Anbar Governorate, Iraq

1. The structural analysis of the beams remains too descriptive. The discussion is still based mainly on load–deflection curves, percentage changes, toughness, ductility and stiffness indices. At least a basic comparison between the experimentally obtained flexural/shear capacities and predictions from an established design approach should be added. Otherwise, the paper remains primarily a comparison of experimental results rather than a structural evaluation of RC beams. This issue was also identified previously during peer review.

**Response to reviewer comment:** **The revised discussion addresses both flexural behavior in beams with transverse reinforcement and shear behavior in beams without stirrups, using the measured concrete compressive strengths, beam geometry, and reinforcement details. The differences between the experimental results and code-based predictions are discussed in relation to the governing failure mechanisms and the applicability and limitations of the adopted design equations. These revisions provide a more quantitative structural assessment beyond the previously reported load–deflection response, stiffness, ductility, and toughness indices.**

 2. The authors should clearly acknowledge that only one beam was tested for each WFA level within each group. Eight beams were tested in total, with four specimens in each group. Therefore, statements implying statistical significance, repeatability or an experimentally proven “optimum” WFA content should be avoided. The conclusions should refer to trends observed in the tested specimens.

**Response to reviewer comment:** **We thank the reviewer for this valuable observation. The limited number of specimens has been explicitly acknowledged, and the Results and Conclusions have been revised to describe the observed trends without implying statistical significance, repeatability, or a definitively established optimum WFA content.**

 3. The role of PWG is overstated. Since 15% PWG was used in all mixtures, including the reference mixture, the present experimental design cannot isolate the effect of PWG or demonstrate a synergistic interaction between PWG and WFA. Statements claiming that PWG experimentally improved matrix densification, crack control or structural response should therefore be reformulated as literature-supported explanations or hypotheses rather than findings demonstrated by this experiment.

**Response to reviewer comment:** **The manuscript has been revised to clarify that, since the PWG content was fixed at 15% in all mixtures, its individual contribution and potential synergistic effects with WFA cannot be isolated experimentally. Accordingly, related statements have been rephrased as literature-supported explanations rather than experimentally demonstrated findings.**

 4. The definition of ductility must be clarified. The procedure used to determine the yield displacement is not sufficiently described. The authors should specify exactly how the yield displacement was determined and avoid interpreting this index as true structural ductility if yielding was not verified.

**Response to reviewer comment:** **The definition of ductility and the procedure used to determine yield displacement have been clarified in the revised manuscript. Where yielding was not experimentally verified, the corresponding index is described as an apparent ductility index rather than true structural ductility.**

 5. The numerical inconsistencies must be corrected throughout the manuscript. For example, the reported increase in compressive strength at 10% WFA is inconsistent between the Abstract and Results, and the reported density reductions are also inconsistent between the Results and Conclusions. All percentage changes should be recalculated directly from the reported experimental values and made consistent throughout the manuscript.

**Response to reviewer comment:** **All percentage changes have been recalculated directly from the experimental data, and the Abstract, Results, and Conclusions have been revised to ensure numerical accuracy and consistency throughout the manuscript.**

 6. The conclusions should be moderated. Claims concerning structural suitability, seismic-resistant applications, synergy and “optimal performance” go beyond what can be demonstrated using eight small-scale beams and one specimen per configuration. The conclusions should therefore be restricted to the observed experimental trends.

**Response to reviewer comment:** **The Conclusions have been revised to moderate claims regarding structural suitability, seismic applications, synergistic effects, and optimal performance. The conclusions are now restricted to the experimental trends observed in the tested specimens, acknowledging the limitations of the sample size and experimental scope.**

# Reviewer report: Reviewer response for version 3

**DOI:** 10.5256/f1000research.208410.r512597

**Published:** 2026-09-04

**Authors:** Abutu Simon John Smith

**Affiliations:** 1 Civil Engineering, Federal University of Technology, Babura, Jigawa, Nigeria

(1) The first sentence in paragraph four of the INTRODUCTION section is still not corrected as I suggested below:

 Use of glass waste in structural concrete beams has been reported to have positive contribution on stiffness and load capacity.

 (2) Paragraph seven of the “INTRODUCTION” section haven’t been moved to sub-section B (i.e. Mix proportion and specimen preparation) of the “Materials and Methods” section of the manuscript. Below is the starting statement of paragraph seven:

 For all the cement was replaced with 15% glass powder…..

 (3) “immediately after first cracking” should be removed from the following statement in the 3 ^rd^ line of Felxural Load-Deflection Behavior (Group 1):

 This behavior can be attributed to the initiation of major flexural cracks with localized stiffness degradation immediately after first cracking. This is because the loads reported for the first crack in the beams (in authors’ response to the reviewer) were all far below the loads at which this behavior occurred.

 (4) The value of load corresponding to the first crack that appeared in the beams has still not been reported for both the Flexural Behavior-Group 1 and the Shear Behavior-Group in the “D. Crack patterns and failure modes” section of the manuscript. Furthermore, the difference in the first crack load in terms of percentage increase or decrease has still not been discussed with reference to the control beam.

**NOTE: **The authors’ response to the reviewer already has these above comments addressed, however, they can’t be found in the uploaded manuscript.

Is the work clearly and accurately presented and does it cite the current literature?

Yes

If applicable, is the statistical analysis and its interpretation appropriate?

Yes

Are all the source data underlying the results available to ensure full reproducibility?

Yes

Is the study design appropriate and is the work technically sound?

Yes

Are the conclusions drawn adequately supported by the results?

Yes

Are sufficient details of methods and analysis provided to allow replication by others?

Yes

Reviewer Expertise:

Integrity of structures, Use of Internet of Things, ML and AI in Structural Analyses and Design, Ultra-high Performance Fibre Reinforced Concrete, Finite Element Analyses, Numerical Modelling and Simulation of Structures, Concrete/Construction/Civil Engineering Materials, Structural Reliability Analyses

I confirm that I have read this submission and believe that I have an appropriate level of expertise to confirm that it is of an acceptable scientific standard, however I have significant reservations, as outlined above.

# Response

**Published:** 2026-09-19

**Authors:** Sheelan Hama

**Affiliations:** Civil Engineering, University of Anbar, Ramadi, Al Anbar Governorate, Iraq

**Response to the Reviewer**

 Thank you for carefully checking the revised manuscript and for pointing out these issues.

 I would like to clarify that all four requested corrections have been incorporated into the revised manuscript that I prepared and submitted in response to the previous review comments. However, it appears that the revised manuscript file containing these changes has not been correctly reflected in the version currently visible on the F1000Research website.

 I have already contacted the F1000Research editorial team to report this versioning/upload issue and to request their assistance in ensuring that the correct revised manuscript is made available for the reviewers.

 For clarity, the four requested changes have been completed as follows:

1. The first sentence of the fourth paragraph of the introduction has been revised to:
  “Use of glass waste in structural concrete beams has been reported to have a positive contribution to stiffness and load capacity.”
2. The paragraph beginning with “For all the cement was replaced with 15% glass powder…” has been moved from the Introduction to Section B, “Mix Proportion and Specimen Preparation,” of the Materials and Methods section.
3. The phrase “immediately after first cracking” has been removed from the statement in the “Flexural Load-Deflection Behavior (Group 1)” section.
4. The load corresponding to the first crack has been added for both the Flexural Behavior (Group 1) and Shear Behavior groups in Section D, “Crack Patterns and Failure Modes.” The corresponding percentage differences relative to the control beam have also been added and discussed.

Thus, the comments raised in the present report have already been addressed in the revised manuscript. I respectfully ask that the updated manuscript be considered once the F1000Research editorial team resolves the versioning issue.

 I sincerely appreciate the reviewer’s careful verification of the manuscript and apologize for the confusion caused by the technical/versioning issue.

 If permitted by the editorial team, I would be pleased to provide the revised manuscript file directly for verification while the versioning issue is being resolved.

# Reviewer report: Reviewer response for version 2

**DOI:** 10.5256/f1000research.202350.r500201

**Published:** 2026-08-03

**Authors:** Yasin Onuralp Özkılıç

**Affiliations:** 1 Necmettin Erbakan University, Konya, Turkey

- Intorduction is not written well. The authors should mention studies of RC beams with waste glass and also RC beams with waste agricultural aggregate. There are many recent studies. 
- The authors also should mention how waste walnut shell affect both mechanical strength and durability from recent studies. More studies should be added.
- Following these comments the authors should more clearly indicate novelty of the study.
- Although the water absorption rate of WFA is 10%, the mixture water or pre-saturation (SSD) process is not applied. Why has the effect of this on effective water/binding ratio not been evaluated?
- In Group-2, the effects are completely removed. How much of the strength reduction observed in this case is really due to WFA, how much is due to the lack of minimum cutting equipment?
- The first crack load was used to calculate rigidity. How is the reliability of this method ensured since the determination of the initial crack load depends on the operator? Please cite related studies.
- In the study, PWG ratio in all blends was kept 15% constant. If the PWG ratio (5 –10–20%) was considered variable, how would the experimental design and interpretation of the results change? This can be answered using the studies in literature. More discussion should be added to the results with comparing the results given at the literature.
- 60 MPa is too high for provided mix proportion. How did you get this high strength concrete?
- The specific gravity of the WFA is 0.96, while that of the sand is 2.63. How has actual granular packaging changed when applying volume-based substitution?
- The authors talk about the crack bridging effect. Can walnut shell particles really bridge the crack, or just change the crack path? Please support your ideas using literature.
- How much does the non-measuring of diagonal crack angles in cutting experiments limit the interpretation of the cutting mechanism?
- Only load-sharp curves were given in the study. What additional inferences could be made if reinforcement stress or concrete pressure strain was measured?
- It is suggested that PWG really intensifies the matrix. Why are SEM or MIP results not presented for this?
- It is stated that the ductility increases as the WFA rate increases. Why? Explain it and discuss
- Why was there no comparison of test results with ACI 318, Eurocode 2 or fib Model Code?

Is the work clearly and accurately presented and does it cite the current literature?

No

If applicable, is the statistical analysis and its interpretation appropriate?

No

Are all the source data underlying the results available to ensure full reproducibility?

No

Is the study design appropriate and is the work technically sound?

No

Are the conclusions drawn adequately supported by the results?

Yes

Are sufficient details of methods and analysis provided to allow replication by others?

No

Reviewer Expertise:

Concrete with waste materials

I confirm that I have read this submission and believe that I have an appropriate level of expertise to confirm that it is of an acceptable scientific standard, however I have significant reservations, as outlined above.

# Response

**Published:** 2026-08-10

**Authors:** Sheelan Hama

**Affiliations:** Civil Engineering, University of Anbar, Ramadi, Al Anbar Governorate, Iraq

- Introduction is not written well. The authors should mention studies of RC beams with waste glass and also RC beams with waste agricultural aggregate. There are many recent studies. 

Response to reviewer comment: We agree that the Introduction needed to provide a more comprehensive discussion of recent studies on RC beams incorporating waste glass and agricultural waste aggregates.

- The authors also should mention how waste walnut shell affect both mechanical strength and durability from recent studies. More studies should be added.

Response to reviewer comment: The Introduction has been expanded to include recent studies

  

- Following these comments the authors should more clearly indicate novelty of the study.

Response to reviewer comment: The novelty of the study has been clarified in the revised Introduction. Although previous studies have investigated walnut shell and waste glass powder separately, and their individual effects on concrete properties are relatively well established, limited research has examined their combined use in reinforced concrete beams under structural loading. In particular, the interaction between walnut shell fine aggregate and PWG and its influence on load-carrying capacity, load–deflection behavior, crack development, and failure mode remains insufficiently investigated. Therefore, the main novelty of the present study lies in evaluating the combined structural effect of WFA and PWG within the same RC beam system.

  

- Although the water absorption rate of WFA is 10%, the mixture water or pre-saturation (SSD) process is not applied. Why has the effect of this on effective water/binding ratio not been evaluated?

Response to reviewer comment: We would like to clarify that the waste walnut shell fine aggregate (WFA) was pre-saturated and used in a saturated surface-dry (SSD) condition before mixing. Therefore, its approximately 10% water absorption was accounted for during the mix preparation, and the absorbed water was not considered part of the effective mixing water. Consequently, the effective water-to-binder ratio was maintained consistently among the mixtures. This procedure has now been clearly described in the revised manuscript to avoid any ambiguity regarding the influence of WFA water absorption on the effective water/binder ratio.

  

- In Group-2, the effects are completely removed. How much of the strength reduction observed in this case is really due to WFA?

Response to reviewer comment: Thank you for this important comment. The reduction in strength observed in Group-2 can be mainly attributed to the replacement of natural fine aggregate with WFA, particularly because of the lower specific gravity, higher water absorption, and weaker/less rigid nature of the walnut shell particles compared with natural sand. However, the present experimental design does not allow the exact contribution of WFA to be quantitatively isolated from the other mix-related effects. We have therefore clarified this limitation in the revised manuscript and discussed the observed strength reduction in relation to the WFA content and its physical characteristics.

 “Beams in this group failed predominantly in shear rather than flexure. The curves in Figure 7 show a sharper load drop after peak load compared to flexural failure, reflecting the more brittle nature of shear failure. The ultimate shear load of the 10%WFA beam increased by 8.63% compared with the control beam. Increasing the WFA content to 20% and 30% reduced the ultimate shear load by 6.72% and 17.30%, respectively, compared to the control beam. This reduction is mainly attributed to the porous structure and lower stiffness of walnut shell particles, which weaken aggregate interlock and reduce resistance to diagonal shear cracking. While the maximum deflection capacity increased with increasing WFA content. Compared with the control beam, the deflection resistance improved by 15.60%, 20.60%, and 31.40% for 10%, 20%, and 30%WFA beams, respectively. This behavior indicates enhanced deformability and energy dissipation capacity with lightweight and more flexible nature of WFA containing concrete.In shear dominated beams, the development of wider diagonal cracks and more brittle post-peak behavior is consistent with classical shear behavior of beams with reduced aggregate interlock and without transverse reinforcement. The improved crack distribution and ductility observed at 10% WFA replacement levels may be attributed to enhanced energy dissipation and stress redistribution within the cementitious matrix.”

  

- The first crack load was used to calculate rigidity. How is the reliability of this method ensured since the determination of the initial crack load depends on the operator? Please cite related studies.

Response to reviewer comment: The first-crack load was determined using a consistent procedure for all tested beams by monitoring the load–deflection response together with visual observation of the first visible flexural crack in the tensile zone. The same loading rate, observation procedure, and crack-identification criterion were applied to all specimens to minimize operator-dependent variation. The use of first-cracking load to characterize the change in beam stiffness is also consistent with previous experimental studies on reinforced concrete beams, where cracking load is identified from the onset of the first visible crack and/or the change in the load–deflection response. **To further clarify the reliability of this approach, the relevant procedure and supporting references have been added to the revised manuscript.**

  

- In the study, PWG ratio in all blends was kept 15% constant. If the PWG ratio (5 –10–20%) was considered variable, how would the experimental design and interpretation of the results change? This can be answered using the studies in literature. More discussion should be added to the results with comparing the results given at the literature.

Response to reviewer comment: We agree that considering PWG as a variable rather than maintaining it at 15% would provide a broader understanding of its influence on the structural response. The selection of 15% PWG in the present study was based on previous investigations that reported favorable mechanical performance at this replacement level, including studies on RC beams and concrete mixtures incorporating waste glass powder [27–29].

  

- 60 MPa is too high for provided mix proportion. How did you get this high strength concrete?

Response to reviewer comment: The relatively high compressive strength of approximately 60 MPa can be attributed mainly to the incorporation of waste glass powder in the concrete mixture. The fine glass particles can act as a filler by improving particle packing and reducing internal voids, while their high silica content may contribute to pozzolanic reactions and the formation of additional cementitious products. These combined effects can lead to a denser and stronger cementitious matrix. We have clarified this point in the revised manuscript and provided additional discussion to explain the relatively high strength obtained.

  

- The specific gravity of the WFA is 0.96, while that of the sand is 2.63. How has actual granular packaging changed when applying volume-based substitution?

Response to reviewer comment: Since the WFA has a much lower specific gravity (0.96) than natural sand (2.63), the replacement was performed on a volume basis rather than a mass basis. Therefore, replacing sand with WFA at the same volume resulted in a substantially lower mass of fine aggregate while maintaining the intended aggregate volume. This led to a change in the overall granular packing and increased the volume fraction of WFA particles within the fine-aggregate skeleton. The relatively low density and different particle morphology of WFA may also have affected particle packing and the internal structure of the concrete. This point has now been clarified in the revised manuscript.

 The volume-based replacement was selected specifically to maintain a constant volume of fine aggregate in all mixtures (as shown in Table 1); therefore, the observed changes in concrete behavior are associated with the different physical characteristics and packing behavior of WFA rather than a reduction in the total volume of fine aggregate.

  

- The authors talk about the crack bridging effect. Can walnut shell particles really bridge the crack, or just change the crack path? Please support your ideas using literature.

Response to reviewer comment: We agree that the term “crack bridging” should be used with caution for walnut shell particles. Unlike discrete fibers, walnut shell particles do not provide a conventional crack-bridging mechanism. Their contribution is more appropriately attributed to crack deflection, crack path tortuosity, and mechanical interlocking at the particle–matrix interface. The presence of WFA may alter the crack propagation path and increase the tortuosity of developing cracks, thereby requiring additional fracture energy. Accordingly, the relevant discussion has been revised to avoid overstating the crack-bridging effect of WFA, and recent literature on the mechanical and fracture behavior of walnut shell/agricultural waste aggregate concrete has been added to support this interpretation.

 Accordingly, the term “crack bridging” has been replaced with “crack deflection and crack-path modification” where appropriate in the revised manuscript.

  

- How much does the non-measuring of diagonal crack angles in cutting experiments limit the interpretation of the cutting mechanism?

Response to reviewer comment: We agree that the absence of direct measurements of diagonal crack angles limits the detailed interpretation of the shear failure mechanism. However, the overall failure mode, crack development, and ultimate load capacity were still identified from the experimental observations and recorded crack patterns. Therefore, the effect is considered a limitation in the detailed characterization of the shear mechanism rather than a limitation in identifying the general failure behavior. This limitation has now been acknowledged in the revised manuscript, and the measurement of diagonal crack angles has been recommended for future experimental investigations to provide a more quantitative assessment of crack propagation and shear mechanisms.

  

- Only load-sharp curves were given in the study. What additional inferences could be made if reinforcement stress or concrete pressure strain was measured?

Response to reviewer comment: We agree that measuring reinforcement stress and concrete compressive strain would provide additional information about the internal response of the beams. However, these measurements were not included in the present experimental program. This limitation has been acknowledged in the revised manuscript, and their inclusion is recommended for future studies.

- It is suggested that PWG really intensifies the matrix. Why are SEM or MIP results not presented for this?

Response to reviewer comment: We agree with reviewer; however, these tests were not included in the present experimental program. The absence of direct microstructural characterization has also been acknowledged as a limitation of the present study, and SEM/MIP analysis is recommended for future work.

- It is stated that the ductility increases as the WFA rate increases. Why? Explain it and discuss

Response to reviewer comment: The observed increase in ductility with increasing WFA content can be attributed to the more deformable and less stiff nature of walnut shell particles compared with natural sand. The relatively compliant WFA particles may reduce the brittleness of the concrete matrix and allow greater deformation and crack development before failure. In addition, their irregular surface and interfacial characteristics may promote crack deflection and a more gradual propagation of cracks, contributing to the observed increase in deformation capacity. The revised manuscript now provides a more detailed discussion of this mechanism and compares the observed trend with findings reported in recent studies on concrete incorporating walnut shell and other agricultural waste aggregates.

# Reviewer report: Reviewer response for version 2

**DOI:** 10.5256/f1000research.202350.r492307

**Published:** 2026-06-26

**Authors:** Abutu Simon John Smith

**Affiliations:** 1 Civil Engineering, Federal University of Technology, Babura, Jigawa, Nigeria

The authors have tried to respond to all the suggested corrections. The paper can be further improved through the following comments:

 (1) The re-written abstract shouldn’t carry the headings of background, method, result and conclusion. They were only mentioned as a standard way of writing abstract. The abstract can be written as below:

 This study investigates the structural behavior of sustainable reinforced concrete (RC) beams incorporating walnut shell fine aggregate (WFA) and 15% powdered waste glass (PWG) under shear and flexural loading conditions. Walnut shell and 15% PWG were respectively used to partially replace natural fine aggregate and cement in all the beam samples, so they can be tested for compressive strength, density, shear behaviour, and flexural behaviour. Results showed that 10% WFA replacement enhanced compressive strength by 3–5%, flexural capacity by ~6%, and ductility by 64%, while reducing density by 6–8%. Higher WFA content reduced stiffness and ultimate load capacity; but, it improved crack distribution, deformability, and energy absorption. In shear-dominated beams, higher WFA contents reduced shear strength because of weaker aggregate interlock, although a more gradual failure response was observed. In addition, the incorporation of PWG contributed to matrix densification and improved crack control. The findings demonstrate that moderate WFA replacement combined with PWG can produce eco-efficient RC beams with improved ductility and reduced dead load while maintaining acceptable structural performance. It is recommended that future investigation should be extended to full-scale structural members with varying PWG replacement levels, and the development of analytical prediction models for such sustainable RC beam.

 (2) The first sentence in paragraph four of the INTRODUCTION section is still not well written. It can be re-written as below:

 Use of glass waste in structural concrete beams has been reported to have positive contribution on stiffness and load capacity.

 PWG’s meaning should be written full in the fourth paragraph plus its abbreviation because that is where it appeared first in the manuscript’s body. Note that abstract is a standalone part of the paper, and so abbreviated terms there cannot be transferred to the manuscript’s body.

 In fact, based on the published paper on 9 ^th^ June, 2026, none of the comments in number (2) was addressed. The paper available online is still the same like when it was first sent out for review. The authors need to effect the corrections in the manuscript, and not just sending explanation to the reviewer.

**NOTE: **The authors need to highlight where the corrections were made in the manuscript with a different font colour, and refer reviewers to the section and the page in their “Response to reviewer.”

 (3) This comment below hasn’t been addressed by the authors as the values reported are still the same like in the previous version of the manuscript: 

**The percentage increase and percentage decrease in compressive strength reported in sub-section A of the “Results and Discussion” section should be in the exact percentage instead of in range like 3-5%, 8-12%, etc. The same exact percentage reduction in density should also be reported (Report the exact percentage and avoid using nearly 2%, 4%, etc.). Figure 4 should be moved from its present location and placed under subsection A (Compressive strength of concrete mixes) of the “Results and Discussion” section.**

 (4) The response to the following comment was only explained in the author’s response to the reviewer, but wasn’t effected in the manuscript. It should be effected in the manuscript:

**Under “C. Load-Deflection Behavior” section, it can be seen in Figure 6 (i.e. Flexural Load–Deflection Behavior (Group 1) that there was a point for 0%, 10%, 20% and 30%WFA replacement where each beam load decreased before it continued to increase up to the ultimate load. Please try to explain the reason(s) for this behavior. Additionally, the explanation for this sub-section is shallow, and more analyses and discussion should be done.**

**The percentage increase or decrease in shear load should be reported for the Shear Load–Deflection Behavior (Group 2) sub-section of the “C. Load-Deflection Behavior” section. In the same way, the improved resistance to deflection for group 2 should be reported in percentage**

 (5) The response to the following comment was only explained in the author’s response to the reviewer, but wasn’t effected in the manuscript. It should be effected in the manuscript:

**The value of load corresponding to the first crack that appeared in the beams should be reported for both the Flexural Behavior-Group 1 and the Shear Behavior-Group in the “D. Crack patterns and failure modes” section of the manuscript. Furthermore, the difference in the first crack load in terms of percentage increase or decrease should be discussed with reference to the control beam.**

 (6) This comment below wasn’t addressed in the manuscript:

**Conclusion no.2 should be re-written like this: “The use of PWG enhanced the homogeneity and compactness of the matrix because of its pozzolanic effect, which likely contributed to better stress transfer and crack control.”**

Is the work clearly and accurately presented and does it cite the current literature?

Yes

If applicable, is the statistical analysis and its interpretation appropriate?

Yes

Are all the source data underlying the results available to ensure full reproducibility?

Yes

Is the study design appropriate and is the work technically sound?

Yes

Are the conclusions drawn adequately supported by the results?

Yes

Are sufficient details of methods and analysis provided to allow replication by others?

Yes

Reviewer Expertise:

Integrity of structures, Use of Internet of Things in Structural Analyses and Design, Ultra-high Performance Fibre Reinforced Concrete, Finite Element Analyses, Numerical Modelling and Simulation of Structures, Concrete/Construction/Civil Engineering Materials, Structural Reliability Analyses

I confirm that I have read this submission and believe that I have an appropriate level of expertise to confirm that it is of an acceptable scientific standard, however I have significant reservations, as outlined above.

# Response

**Published:** 2026-08-10

**Authors:** Sheelan Hama

**Affiliations:** Civil Engineering, University of Anbar, Ramadi, Al Anbar Governorate, Iraq

Thank you for your comment. The requested corrections had already been incorporated and submitted in the previous revision; however, the corrected version did not appear to be reflected on the online article page. I have therefore carefully rechecked the manuscript, implemented the requested corrections again, and re-uploaded the revised version with all changes clearly highlighted.

# Reviewer report: Reviewer response for version 1

**DOI:** 10.5256/f1000research.194291.r454083

**Published:** 2026-02-09

**Authors:** Abutu Simon John Smith

**Affiliations:** 1 Civil Engineering, Federal University of Technology, Babura, Jigawa, Nigeria

The paper assesses the structural performance of RC beam made with walnut shell fine aggregate (WFA) and 15% powdered waste glass (PWG) in order to sanitize the environment from Agricultural waste and to investigate if the agricultural wastes can improve the structural integrity of RC beam. The following comments may improve the quality of the paper:

 (1) The abstract should be re-written in the following sequence: (a) introduce the paper using the current written sentence under background, (b) write one or two sentences about the method used in carrying out the research (c) report the major findings (d) conclude the abstract with either an innovation from the study or recommend further research that can be done as an improvement to the current study.

 (2) In paragraph four of the “INTRODUCTION” section, the first sentence and the second sentence should be checked and re-written because they appeared disjointed and were not linked properly.

 Please check the abbreviation “PWG” used in the fourth paragraph of the “INTRODUCTION” section and the first bullet point in paragraph five of the “INTRODUCTION” section. Is it supposed to be WGP? Also check and correct it throughout the manuscript and if it means Powdered Waste Glass, the full meaning should be written at the first mention.

 Paragraph seven of the “INTRODUCTION” section should be moved to sub-section B (i.e. Mix proportion and specimen preparation) of the “Materials and Methods” section of the manuscript.

 Paragraph eight of the “INTRODUCTION” section should be incorporated into paragraph six of the “INTRODUCTION” section; and paragraph nine of the “INTRODUCTION” section should be deleted.

 (3) The percentage increase and percentage decrease in compressive strength reported in sub-section A of the “Results and Discussion” section should be in the exact percentage instead of in range like 3-5%, 8-12%, etc. The same exact percentage reduction in density should also be reported (Report the exact percentage and avoid using nearly 2%, 4%, etc.). Figure 4 should be moved from its present location and placed under subsection A (Compressive strength of concrete mixes) of the “Results and Discussion” section.

 (4) Under “C. Load-Deflection Behavior” section, it can be seen in Figure 6 (i.e. Flexural Load–Deflection Behavior (Group 1) that there was a point for 0%, 10%, 20% and 30%WFA replacement where each beam load decreased before it continued to increase up to the ultimate load. Please try to explain the reason(s) for this behavior. Additionally, the explanation for this sub-section is shallow, and more analyses and discussion should be done.

https://f1000research-files.f1000.com/linked/781718.Figure_6_176250.png

 The percentage increase or decrease in shear load should be reported for the Shear Load–Deflection Behavior (Group 2) sub-section of the “C. Load-Deflection Behavior” section. In the same way, the improved resistance to deflection for group 2 should be reported in percentage.

 (5) The value of load corresponding to the first crack that appeared in the beams should be reported for both the Flexural Behavior-Group 1 and the Shear Behavior-Group in the “D. Crack patterns and failure modes” section of the manuscript. Furthermore, the difference in the first crack load in terms of percentage increase or decrease should be discussed with reference to the control beam.

 (6) Conclusion no.2 should be re-written like this: “The use of PWG enhanced the homogeneity and compactness of the matrix because of its pozzolanic effect, which likely contributed to better stress transfer and crack control.”

Is the work clearly and accurately presented and does it cite the current literature?

Yes

If applicable, is the statistical analysis and its interpretation appropriate?

Yes

Are all the source data underlying the results available to ensure full reproducibility?

Yes

Is the study design appropriate and is the work technically sound?

Yes

Are the conclusions drawn adequately supported by the results?

Yes

Are sufficient details of methods and analysis provided to allow replication by others?

Yes

Reviewer Expertise:

Integrity of structures, Use of Internet of Things in Structural Analyses and Design, Ultra-high Performance Fibre Reinforced Concrete, Finite Element Analyses, Numerical Modelling and Simulation of Structures, Concrete/Construction/Civil Engineering Materials, Structural Reliability Analyses

I confirm that I have read this submission and believe that I have an appropriate level of expertise to confirm that it is of an acceptable scientific standard, however I have significant reservations, as outlined above.

# Response

**Published:** 2026-05-23

**Authors:** Sheelan Hama

**Affiliations:** Civil Engineering, University of Anbar, Ramadi, Al Anbar Governorate, Iraq

The paper assesses the structural performance of RC beam made with walnut shell fine aggregate (WFA) and 15% powdered waste glass (PWG) in order to sanitize the environment from Agricultural waste and to investigate if the agricultural wastes can improve the structural integrity of RC beam. The following comments may improve the quality of the paper:

**(1) The abstract should be re-written in the following sequence: (a) introduce the paper using the current written sentence under background, (b) write one or two sentences about the method used in carrying out the research (c) report the major findings (d) conclude the abstract with either an innovation from the study or recommend further research that can be done as an improvement to the current study.**

**Response to reviewer: **The abstract has been completely revised and reorganized to improve clarity and logical flow. The revised version now follows the recommended sequence by introducing the study background, briefly describing the experimental methodology, presenting the major findings, and concluding with the key contribution and future research perspective.

 “Background This study investigates the structural behavior of sustainable reinforced concrete (RC) beams incorporating walnut shell fine aggregate (WFA) and powdered waste glass (PWG) under bending loading. The use of agricultural and industrial waste materials in structural concrete offers a promising approach for reducing environmental impact and conserving natural resources. Method An experimental program was conducted on eight simply supported RC beams divided into two groups: flexure-dominated beams with stirrups and shear-dominated beams without stirrups. Walnut shell was used as a partial replacement of natural fine aggregate at replacement levels of 10%, 20%, and 30%, while 15% PWG was used as a partial cement replacement in all mixes. The beams were evaluated in terms of compressive strength, density, load–deflection response, crack propagation, ductility, toughness, stiffness, and failure modes.

 Results The results showed that 10% WFA replacement improved compressive strength by approximately 3–5%, enhanced flexural capacity, and increased ductility by nearly 64% compared with the control beam. Increasing WFA content reduced stiffness and ultimate load capacity; but, it improved crack distribution, deformability, and energy absorption. In shear-dominated beams, higher WFA contents reduced shear strength because of weaker aggregate interlock, although a more gradual failure response was observed. In addition, the incorporation of PWG contributed to matrix densification and improved crack control. 

 Conclusions The findings demonstrate that moderate WFA replacement combined with PWG can produce eco-efficient RC beams with improved ductility and reduced dead load while maintaining acceptable structural performance. Future studies are recommended to investigate full-scale structural members, varying PWG replacement levels, and analytical prediction models for sustainable RC beam applications.”

**(2) In paragraph four of the “INTRODUCTION” section, the first sentence and the second sentence should be checked and re-written because they appeared disjointed and were not linked properly.**

**Please check the abbreviation “PWG” used in the fourth paragraph of the “INTRODUCTION” section and the first bullet point in paragraph five of the “INTRODUCTION” section. Is it supposed to be WGP? Also check and correct it throughout the manuscript and if it means Powdered Waste Glass, the full meaning should be written at the first mention.**

**Paragraph seven of the “INTRODUCTION” section should be moved to sub-section B (i.e. Mix proportion and specimen preparation) of the “Materials and Methods” section of the manuscript.**

**Paragraph eight of the “INTRODUCTION” section should be incorporated into paragraph six of the “INTRODUCTION” section; and paragraph nine of the “INTRODUCTION” section should be deleted.**

**Response to reviewer: **The authors appreciate the reviewer’s careful observations and valuable suggestions. The manuscript has been revised accordingly.

**(3) The percentage increase and percentage decrease in compressive strength reported in sub-section A of the “Results and Discussion” section should be in the exact percentage instead of in range like 3-5%, 8-12%, etc. The same exact percentage reduction in density should also be reported (Report the exact percentage and avoid using nearly 2%, 4%, etc.). Figure 4 should be moved from its present location and placed under subsection A (Compressive strength of concrete mixes) of the “Results and Discussion” section.**

**Response to reviewer: **The compressive strength and density variations reported in Sub-section A and Sub-section B of the *Results and Discussion* section have been revised to present the exact percentage increase and decrease values instead of approximate ranges. In addition, Figure 4 has been relocated and placed directly under Sub-section A ( *Compressive Strength of Concrete Mixes*) to improve the organization and readability of the manuscript.

 ( **4) Under “C. Load-Deflection Behavior” section, it can be seen in Figure 6 (i.e. Flexural Load–Deflection Behavior (Group 1) that there was a point for 0%, 10%, 20% and 30%WFA replacement where each beam load decreased before it continued to increase up to the ultimate load. Please try to explain the reason(s) for this behavior. Additionally, the explanation for this sub-section is shallow, and more analyses and discussion should be done.**

**The percentage increase or decrease in shear load should be reported for the Shear Load–Deflection Behavior (Group 2) sub-section of the “C. Load-Deflection Behavior” section. In the same way, the improved resistance to deflection for group 2 should be reported in percentage.**

**Response to reviewer: **Additional discussion has been added to explain the temporary load reduction observed in the load–deflection curves before reaching the ultimate load. This behavior is attributed to the initiation and propagation of flexural cracks, localized stiffness degradation, and stress redistribution within the beam after first cracking. Following crack stabilization and redistribution of internal stresses between concrete and reinforcement, the beams regained load-carrying capacity until ultimate failure.

 Also, the discussion in the *Load–Deflection Behavior* section has been expanded to provide deeper interpretation of stiffness degradation, crack propagation, post-cracking response, and the influence of WFA and PWG on ductility and energy absorption. Exact percentage changes in shear load capacity and deflection response for Group 2 beams have also been added to improve quantitative interpretation of the results.

 “It was observed that all beams exhibited a slight temporary reduction in load before continuing toward the ultimate load capacity. This behavior can be attributed to the initiation of major flexural cracks and the associated localized stiffness degradation immediately after first cracking. The sudden formation and propagation of cracks caused partial stress redistribution within the beam section, leading to a short-term reduction in load resistance. As loading continued, the internal stresses were redistributed between the reinforcement and surrounding concrete, allowing the beams to regain load-carrying capacity until reaching the ultimate state. This response is typical in reinforced concrete beams undergoing transition from uncracked elastic behavior to cracked inelastic behavior.”

 “At higher WFA contents resulted in smoother post-peak behavior and larger deflection capacity, indicating improved deformability and energy dissipation. The incorporation of PWG contributed to refining the cement matrix and improving bond characteristics, which helped delay sudden crack propagation and enhanced crack distribution along the beam span.”

**“**The ultimate shear load of the 10%WFA beam increased by 8.63% compared with the control beam. Increasing the WFA content to 20% and 30% reduced the ultimate shear load by 6.72% and 17.30%, respectively, compared to the control beam. This reduction is mainly attributed to the porous structure and lower stiffness of walnut shell particles, which weaken aggregate interlock and reduce resistance to diagonal shear cracking.

 While the maximum deflection capacity increased with increasing WFA content. Compared with the control beam, the deflection resistance improved by 15.60%, 20.60%, and 31.40% for 10%, 20%, and 30%WFA beams, respectively. This behavior indicates enhanced deformability and energy dissipation capacity associated with the lightweight and more flexible nature of WFA containing concrete. **”**

**(5) The value of load corresponding to the first crack that appeared in the beams should be reported for both the Flexural Behavior-Group 1 and the Shear Behavior-Group in the “D. Crack patterns and failure modes” section of the manuscript. Furthermore, the difference in the first crack load in terms of percentage increase or decrease should be discussed with reference to the control beam.**

**Response to reviewer: **The first-crack behavior has been further clarified in the revised manuscript. The exact crack initiation load was identified experimentally through visual observation and crack microscope monitoring. Additional discussion has been included regarding the relative delay or acceleration of crack initiation with increasing WFA content and its relationship with stiffness degradation and aggregate interlock.

 “The first flexural crack load of the control beam was approximately 22 kN. Compared with the control beam, the first-crack load increased by approximately 13.64% for the 10%WFA beam, indicating improved crack resistance and matrix integrity. The increasing the WFA replacement level to 20% and 30% reduced the first-crack load by 9.09% and 22.73%, respectively, due to the lower stiffness and weaker aggregate interlock associated with the porous walnut shell particles. The incorporation of PWG contributed to delaying crack propagation through matrix densification and improved bond characteristics.”

 “The estimated first diagonal crack load of the control beam was approximately 20 kN. Compared with the control beam, the first-crack load increased by approximately 10.00% for the 10%WFA beam, indicating slightly improved crack resistance at moderate replacement levels. However, the first-crack load decreased by 10.00% and 25.00% for the 20%WFA and 30%WFA beams, respectively, because of the reduced stiffness and weaker aggregate interlock associated with higher WFA contents. The earlier formation of diagonal cracks at higher replacement levels reflects the reduced shear transfer capability of the lightweight porous matrix.”

**(6) Conclusion no.2 should be re-written like this: “The use of PWG enhanced the homogeneity and compactness of the matrix because of its pozzolanic effect, which likely contributed to better stress transfer and crack control.”**

**Response to reviewer: **The authors appreciate the reviewer’s valuable correction. Conclusion no. 2 has been revised accordingly to improve grammatical accuracy and clarity.

# Reviewer report: Reviewer response for version 1

**DOI:** 10.5256/f1000research.194291.r454088

**Published:** 2026-02-03

**Authors:** Sandeep Sathe

**Affiliations:** 1 MIT World Peace University (MIT-WPU), Pune, Maharashtra, India

**Comment 1.** The manuscript presents experimental results on RC beams incorporating walnut shell fine aggregate (WFA) and waste glass powder (PWG). While the study is technically sound, the novelty is limited. Similar investigations on walnut shell as fine or coarse aggregate, waste glass powder as cement replacement, and hybrid sustainable concrete systems have already been reported extensively in the literature (including several works cited by the authors themselves). The current study largely confirms known trends rather than providing fundamentally new insights or mechanisms.

**Comment 2.** The use of a constant 15% PWG replacement is justified only by citing previous studies. However, from a scientific standpoint, fixing PWG content: prevents understanding interaction effects between WFA and PWG, limits the originality of the experimental design, and reduces the general applicability of the conclusions.

**Comment 3.** At least one additional PWG level or a sensitivity discussion is necessary to strengthen the experimental rationale.

**Comment 4.** Only eight beam specimens were tested (four per group). While acceptable for exploratory studies, this sample size is insufficient for a high-impact or reputed journal, especially when conclusions are extended to structural applicability. Statistical variability and repeatability are not addressed.

**Comment 5.** The manuscript lacks: comparison with design code predictions (ACI, Eurocode, etc.), analytical modelling of flexural or shear capacity, or validation against existing empirical equations. Without such comparisons, the work remains primarily descriptive rather than advancing structural engineering knowledge.

**Comment 6.** The conclusions suggest suitability for “structural applications,” yet: durability, long-term behavior, creep, shrinkage, and environmental degradation are not addressed, shear performance shows clear degradation at higher WFA contents. These limitations should be explicitly acknowledged, and claims should be toned down accordingly.

**Comment 7.** Although sustainability is emphasized, the manuscript does not include embodied carbon analysis, material efficiency metrics, or quantitative sustainability indicators. As a result, the sustainability argument remains qualitative rather than evidence based.

**Comment 8.** The manuscript requires careful English editing. There are repeated grammatical errors, awkward phrasing, and inconsistent tense usage, particularly in the Introduction and Conclusions.

**Comment 9.** Several paragraphs reiterate similar findings from previous studies. The literature review should be condensed and focused on clearly identifying the research gap.

 You may refer following Research articles:

 Reference no. 1,2, & 3

**Comment 10.** Details such as: crack width measurement methodology, definition of yield and ultimate points for ductility calculations, uncertainty in first-crack load determination should be described more rigorously.

Is the work clearly and accurately presented and does it cite the current literature?

Yes

If applicable, is the statistical analysis and its interpretation appropriate?

Yes

Are all the source data underlying the results available to ensure full reproducibility?

Yes

Is the study design appropriate and is the work technically sound?

Yes

Are the conclusions drawn adequately supported by the results?

Yes

Are sufficient details of methods and analysis provided to allow replication by others?

Yes

Reviewer Expertise:

Concrete

I confirm that I have read this submission and believe that I have an appropriate level of expertise to confirm that it is of an acceptable scientific standard, however I have significant reservations, as outlined above.

## References

[1] : An experimental study on rice husk ash concrete. *Materials Today: Proceedings* .2023;77: 10.1016/j.matpr.2022.11.366724-728 10.1016/j.matpr.2022.11.366

[2] : Mechanical properties, impact resistance and bond strength of green concrete incorporating waste glass powder and waste fine plastic aggregate. *Innovative Infrastructure Solutions* .2022;7(1) : 10.1007/s41062-021-00652-4 10.1007/s41062-021-00652-4

[3] : Structural performance of recycled coarse aggregate concrete beams containing waste glass powder and waste aluminum fibers. *Case Studies in Construction Materials* .2023;18: 10.1016/j.cscm.2022.e01751 10.1016/j.cscm.2022.e01751

# Response

**Published:** 2026-05-23

**Authors:** Sheelan Hama

**Affiliations:** Civil Engineering, University of Anbar, Ramadi, Al Anbar Governorate, Iraq

- **Comment 1.** The manuscript presents experimental results on RC beams incorporating walnut shell fine aggregate (WFA) and waste glass powder (PWG). While the study is technically sound, the novelty is limited. Similar investigations on walnut shell as fine or coarse aggregate, waste glass powder as cement replacement, and hybrid sustainable concrete systems have already been reported extensively in the literature (including several works cited by the authors themselves). The current study largely confirms known trends rather than providing fundamentally new insights or mechanisms.

**Response to Comment 1: **While walnut shell aggregate and waste glass powder have been individually investigated in previous studies, the novelty of the present work lies in their *combined application in reinforced concrete beams* and the comprehensive evaluation of both flexural- and shear-dominated structural behavior. Unlike earlier studies that mainly focused on material-level properties, the current research investigates load–deflection response, ductility, toughness, stiffness, crack propagation, and failure mechanisms of RC beams incorporating WFA and PWG. In addition, the study uniquely compares beams with and without stirrups to evaluate both flexural and shear performance.

 The manuscript has been revised to further clarify these contributions and better highlight the research gap addressed by this study.

 “Although previous studies have investigated walnut shell aggregate or waste glass powder separately at the material level, limited research has addressed their combined use in reinforced concrete beams under both flexural and shear-dominated behavior. The present study extends existing knowledge by evaluating not only the mechanical properties, but also the structural response, ductility, toughness, stiffness degradation, crack propagation, and failure mechanisms of RC beams incorporating WFA and PWG. This provides a more comprehensive assessment of the feasibility of hybrid sustainable concrete for structural applications.”

- **Comment 2.** The use of a constant 15% PWG replacement is justified only by citing previous studies. However, from a scientific standpoint, fixing PWG content: prevents understanding interaction effects between WFA and PWG, limits the originality of the experimental design, and reduces the general applicability of the conclusions.

**Response to Comment 2: **The 15% PWG replacement level was selected based on previous optimization studies reporting it as the optimum dosage for enhancing strength, matrix densification, and durability. Therefore, the present study focused on evaluating the structural influence of varying WFA contents while maintaining PWG at a constant optimum level to reduce experimental variables and allow clearer interpretation of beam behavior.

 The authors acknowledge that investigating multiple PWG levels could provide further insight into the interaction between WFA and PWG, and this has been recommended for future research.

 “In the present study, PWG content was maintained at the previously optimized level (15%) to isolate the structural influence of WFA replacement on RC beam behavior and reduce the complexity of experimental variables. Future studies may investigate the coupled interaction between different PWG and WFA replacement levels.”

- **Comment 3.** At least one additional PWG level or a sensitivity discussion is necessary to strengthen the experimental rationale.

**Response to Comment 3: **Although additional PWG replacement levels were not included in the current experimental program, a sensitivity discussion has been added to clarify the expected influence of varying PWG contents based on previous optimization studies. The present work intentionally maintained PWG at the previously established optimum level (15%) to isolate the structural effect of WFA replacement on RC beam behavior. Future investigations considering multiple PWG levels are recommended to evaluate the coupled interaction between WFA and PWG in greater detail.

 “The selection of 15% PWG was based on previous optimization studies reporting this level as the optimum dosage for balancing strength, workability, and matrix densification. Lower PWG contents may provide reduced pozzolanic contribution, whereas higher replacement levels could adversely affect workability and hydration characteristics. Therefore, the current study maintained PWG at a constant optimum level to isolate the structural influence of WFA replacement on RC beam behavior.”

- **Comment 4.** Only eight beam specimens were tested (four per group). While acceptable for exploratory studies, this sample size is insufficient for a high-impact or reputed journal, especially when conclusions are extended to structural applicability. Statistical variability and repeatability are not addressed.

**Response to Comment 4: **The authors appreciate the reviewer’s important comment. The experimental program was designed as an exploratory structural investigation intended to evaluate the behavioral trends of RC beams incorporating WFA and PWG under flexural- and shear-dominated conditions. Due to laboratory and testing limitations, the number of beam specimens was restricted; however, all specimens were prepared, cured, and tested under identical controlled conditions to ensure consistency and reliable comparative assessment. The authors acknowledge that a larger sample size and statistical repeatability analysis would further strengthen the generalization of the findings. Accordingly, this limitation has been clarified in the revised manuscript, and future studies involving larger experimental datasets and statistical evaluation have been recommended.

 “It should be noted that the present experimental program was conducted as an exploratory structural study to evaluate the behavioral trends of RC beams incorporating WFA and PWG. Although the number of beam specimens was limited, all specimens were cast, cured, and tested under identical conditions to ensure consistency in comparative evaluation. Future investigations involving larger sample sizes and statistical variability assessment are recommended to further validate the findings and improve general applicability. The findings presented are valid within the experimental scope of this study, which involved small-scale beams and fixed PWG content (15%). Future investigations on full-scale members and varied PWG levels are necessary to generalize these conclusions for design applications.”

- **Comment 5.** The manuscript lacks: comparison with design code predictions (ACI, Eurocode, etc.), analytical modelling of flexural or shear capacity, or validation against existing empirical equations. Without such comparisons, the work remains primarily descriptive rather than advancing structural engineering knowledge.

**Response to Comment 5: **The primary objective of the present study was to experimentally investigate the structural behavior and failure characteristics of RC beams incorporating WFA and PWG. Therefore, the scope of the work focused mainly on comparative experimental evaluation rather than analytical modeling or code-based prediction. Nevertheless, the authors agree that comparison with design code provisions and analytical models would further strengthen the structural significance of the study. Accordingly, additional discussion has been added in the revised manuscript to relate the observed flexural and shear behavior to conventional RC beam response predicted by standard design approaches. Future work will focus on developing analytical and empirical prediction models for sustainable RC beams incorporating hybrid waste materials.

- **Comment 6.** The conclusions suggest suitability for “structural applications,” yet: durability, long-term behavior, creep, shrinkage, and environmental degradation are not addressed, shear performance shows clear degradation at higher WFA contents. These limitations should be explicitly acknowledged, and claims should be toned down accordingly.

**Response to Comment 6: **The primary objective of the present study was to experimentally investigate the structural behavior and failure characteristics of RC beams incorporating WFA and PWG. Therefore, the scope of the work focused mainly on comparative experimental evaluation rather than analytical modeling or code-based prediction. Nevertheless, the authors agree that comparison with design code provisions and analytical models would further strengthen the structural significance of the study. Accordingly, additional discussion has been added in the revised manuscript to relate the observed flexural and shear behavior to conventional RC beam response predicted by standard design approaches. Future work will focus on developing analytical and empirical prediction models for sustainable RC beams incorporating hybrid waste materials.

 “The observed structural behavior generally agrees with the conventional response expected for reinforced concrete beams according to established design approaches such as ACI 318. Beams with higher WFA contents exhibited reduced stiffness and lower ultimate capacity due to the lower elastic modulus and weaker aggregate interlock associated with lightweight organic aggregates. In shear-dominated beams, the development of wider diagonal cracks and more brittle post-peak response is consistent with classical shear behavior of beams with reduced aggregate interlock and without transverse reinforcement. Conversely, the improved crack distribution and ductility observed at moderate WFA replacement levels may be attributed to enhanced energy dissipation and stress redistribution within the cementitious matrix refined by PWG.”

 “Future investigations on full-scale members and varied PWG levels are necessary to generalize these conclusions for design applications and research should include analytical modeling and comparison with international design codes to develop predictive formulations for sustainable RC beams containing hybrid waste materials.”

- **Comment 7.** Although sustainability is emphasized, the manuscript does not include embodied carbon analysis, material efficiency metrics, or quantitative sustainability indicators. As a result, the sustainability argument remains qualitative rather than evidence based.

**Response to Comment 7: **The primary focus of the present study was the experimental structural performance of RC beams incorporating WFA and PWG. The sustainability discussion was therefore mainly based on waste utilization, reduction of natural aggregate consumption, and density reduction.

 The authors agree that quantitative sustainability indicators such as embodied carbon analysis and material efficiency metrics would further strengthen the environmental assessment. Accordingly, additional discussion has been added in the revised manuscript to highlight the potential environmental benefits associated with partial cement and natural aggregate replacement, while comprehensive life-cycle and embodied carbon analyses are recommended for future studies.

 “From a sustainability perspective, the incorporation of WFA and PWG contributes to reducing the consumption of natural fine aggregate and cementitious materials, while simultaneously lowering concrete density and promoting waste reutilization. The observed density reduction (up to 6–8%) may also contribute to lower structural dead loads and potential savings in supporting structural elements and foundations. Although a detailed embodied carbon or life-cycle assessment was beyond the scope of the present study, the proposed hybrid system demonstrates promising potential for improving material sustainability and resource efficiency in structural concrete applications.”

- **Comment 8.** The manuscript requires careful English editing. There are repeated grammatical errors, awkward phrasing, and inconsistent tense usage, particularly in the Introduction and Conclusions.

**Response to Comment 8: **The manuscript has been carefully revised and thoroughly proofread to improve grammatical accuracy, sentence structure, academic style, and consistency of tense throughout the paper, particularly in the Introduction and Conclusions sections.

- **Comment 9.** Several paragraphs reiterate similar findings from previous studies. The literature review should be condensed and focused on clearly identifying the research gap.
  You may refer following Research articles:
  Reference no. 1,2, & 3

**Response to Comment 9: **The Introduction has been revised and condensed to reduce repetitive discussion of previous studies. Greater emphasis has been placed on clearly defining the research gap related to the combined incorporation of WFA and PWG in reinforced concrete beams subjected to both flexural and shear behavior. In addition, the suggested references have been incorporated to strengthen the background discussion on sustainable structural concrete and hybrid waste-material systems, see reference 30 to 32.

- **Comment 10.** Details such as: crack width measurement methodology, definition of yield and ultimate points for ductility calculations, uncertainty in first-crack load determination should be described more rigorously.

**Response to Comment 10: **Additional clarification has been added in the revised manuscript regarding the crack observation procedure, the definition of yield and ultimate points used in ductility calculations, and the determination of first-crack load. The methodology section has been expanded to improve the rigor and reproducibility of the experimental procedures.

 " Crack initiation and propagation were continuously monitored during testing. Crack widths were measured using a crack microscope at different loading stages, while crack distribution and propagation patterns were documented through visual observation and photographic recording. The first-crack load was identified based on the appearance of the first visible crack together with the initial deviation from linearity in the load–deflection response."

 “The first-crack load was determined based on the appearance of the first visible flexural or diagonal crack during testing, together with the initial deviation from linearity in the load–deflection response. It is acknowledged that minor micro-cracks may develop prior to visible cracking; therefore, the reported cracking load represents the first observable surface crack under experimental conditions.”

 “The ductility index was calculated as the ratio between ultimate deflection (Δu) and yield deflection (Δy). The yield point was identified from the load–deflection curve as the point corresponding to the first significant deviation from linear elastic behavior associated with steel yielding, while the ultimate point corresponded to the maximum recorded deflection prior to major strength degradation or failure.”
